# CRISPR/Cas9-Induced Mutagenesis Corroborates the Role of Transportin-SR2 in HIV-1 Nuclear Import

**DOI:** 10.1128/Spectrum.01336-21

**Published:** 2021-10-06

**Authors:** Julie Janssens, Jolien Blokken, Yulia Lampi, Flore De Wit, Irena Zurnic Bonisch, Ivan Nombela, Paulien Van de Velde, Barbara Van Remoortel, Rik Gijsbers, Frauke Christ, Zeger Debyser

**Affiliations:** a Laboratory of Molecular Virology and Gene Therapy, Department of Pharmaceutical and Pharmacological Sciences, KU Leuven, Leuven, Flanders, Belgium; Fundacio IrsiCaixa

**Keywords:** CRISPR-Cas9, HIV-1 nuclear import, transportin-SR2, human immunodeficiency virus

## Abstract

To infect nondividing cells, HIV-1 needs to cross the nuclear membrane. The importin transportin-SR2 (TRN-SR2 or transportin-3) has been proposed to mediate HIV-1 nuclear import, but the detailed mechanism remains unresolved. The direct interaction of TRN-SR2 with HIV-1 integrase (IN) has been proposed to drive HIV-1 nuclear import. Alternatively, TRN-SR2 may play an indirect role by mediating nuclear import of cleavage and polyadenylation specificity factor 6 (CPSF6). To unravel the role of TRN-SR2, we designed CRISPR/Cas9 guide RNAs targeting different exons of *TNPO3*. Although this approach failed to generate full knockouts, monoallelic knockout clones were generated with indel mutations. HIV-1 replication was hampered in those clones at the level of HIV-1 nuclear import without an effect on the cellular distribution of the TRN-SR2 cargoes CPSF6 or alternative splicing factor1/pre-mRNA splicing factor SF2 (ASF/SF2). Recombinant ΔV_105_ TRN-SR2 expressed in clone 15.15 was 2-fold impaired for interaction with HIV-1 IN and classified as an interaction mutant. Our data support a model whereby TRN-SR2 acts as a cofactor of HIV-1 nuclear import without compromising the nuclear import of cellular cargoes. CRISPR/Cas9-induced mutagenesis can be used as a method to generate interface mutants to characterize host factors of human pathogens.

**IMPORTANCE** Combination antiretroviral therapy (cART) effectively controls HIV-1 by reducing viral loads, but it does not cure the infection. Lifelong treatment with cART is a prerequisite for sustained viral suppression. The rapid emergence of drug-resistant viral strains drives the necessity to discover new therapeutic targets. The nuclear import of HIV-1 is crucial in the HIV-1 replication cycle, but the detailed mechanism remains incompletely understood. This study provides evidence that TRN-SR2 directly mediates HIV-1 nuclear import via the interaction with HIV-1 integrase. The interaction between those proteins is therefore a promising target toward a rational drug design which could lead to new therapeutic strategies due to the bottleneck nature of HIV-1 nuclear import.

## INTRODUCTION

As a lentivirus, human immunodeficiency virus type 1 (HIV-1) is able to infect nondividing cells. The viral preintegration complex (PIC) actively travels through a pore of the nuclear envelope (1, 2) prior to integration of the viral DNA copy in the host genome. Although the exact mechanism remains unknown, PIC components interact with cellular import factors at the nuclear pore complex (NPC) to shuttle the genetic material of the virus into the nucleus (2–4). Among these cellular proteins, nucleoporins (NUPs) (5, 6), cleavage and polyadenylation specificity factor 6 (CPSF6) (7, 8), as well as the β-karyopherin transportin-SR2 (TRN-SR2; transportin-3) (4, 9, 10), have been proposed to support HIV-1 infection. Given the multitude of host factors used in HIV-1 nuclear import, the virus likely uses different pathways (11). Although NUP358 and NUP153 were shown to be the primary docking and translocation factors for PICs upon arrival at the NPC (5), Lee et al. demonstrated that certain HIV-1 capsid (CA) mutations, such as N74D, utilize alternative interactions with NUP155 or NUP160 (11). The abundance of interactions and mechanisms involved in nuclear translocation of the PIC add to the complexity of HIV-1 nuclear import and fuel ongoing discussions on the relative contribution of each individual factor.

Both host and viral determinants mediate HIV-1 nuclear import (4, 8, 12–14). To date, many studies point to HIV-1 CA as the main determinant of nuclear import (11, 15, 16). Still, the fact that CA uncoating is a possible prerequisite for nuclear import may confound the interpretation of experimental results on the role of CA. Moreover, many studies use pleiotropic CA mutants that affect CA stability, uncoating, and dependence on several host factors such as SEC24C (17), NUP153, and CPSF6 (13, 18, 19), complicating interpretation of results. While the importance of TRN-SR2 as a cellular cofactor of HIV-1 replication is generally recognized (4, 9, 10, 13, 20–22), the question of whether TRN-SR2 is directly involved in HIV-1 nuclear import through its interaction with HIV-1 integrase (IN) remains a matter of debate. The same holds true for CPSF6, which has been investigated extensively in recent years, without consensus on the exact mechanism whereby CPSF6 aids HIV-1 trafficking, nuclear import, and/or integration (11, 23–26). Some groups even argue that the block in HIV-1 replication observed after depletion of TRN-SR2 is due to an indirect inhibition of nuclear import mediated by CPSF6 accumulating in the cytoplasm (7). As for TRN-SR2, both a direct role via an interaction with HIV-1 IN (4, 20–22, 27, 28) and an indirect role via interaction with CPSF6 and uncoating of the viral CA have been proposed (7, 8).

TRN-SR2 is the protein resulting from a splice variant encoded by the *TNPO3* gene and belongs to the β-karyopherin family of proteins that shuttle serine-arginine-rich (S/R) and RNA recognition motif (RRM) proteins into the nucleus (29, 30). TRN-SR2 is a flexible protein of solenoid structure, consisting of 20 HEAT (Huntington, elongation factor 3, PR65/A, TOR) repeats, hairpin structures composed of two α-helices connected by a loop (31). TRN-SR2 uses its Arg-rich helix and the charged residues for recognition of phosphorylated SR cargoes (29). To date, crystal structures of TRN-SR2 both by itself or complexed with the cargo alternative splicing factor 1/pre-mRNA splicing factor SF2 (ASF/SF2) or the exchange factor Ran-GTP (Ras-related nuclear protein-GTP) have been resolved (29, 32). Although a small angle X-ray scattering (SAXS) model but not a crystal structure exists for the complex of TRN-SR2 and HIV-1 IN, the interaction interface has been well characterized at amino acid resolution (20, 27). Domains involved are the HEAT repeats 4, 10, and 11 in the N-terminal and central domain of TRN-SR2 (27) and the core and C-terminal domains of HIV-1 IN (20, 28). More specifically, Arg and Lys residues at amino acid positions 262 to 269 in the IN protein were pinpointed as the interaction interface with TRN-SR2 (20, 28). Of note, the interaction with HIV-1 IN occurs at a different site in TRN-SR2 from the bona fide cargo interaction, which may avoid competition for nuclear import.

Previously, our lab showed that TRN-SR2 knockdown (KD) inhibits nuclear import of HIV-1 in cell lines and primary macrophages (4). To ultimately understand the mechanism whereby TRN-SR2 mediates HIV-1 nuclear import, we decided to generate a knockout of this protein using a CRISPR/Cas9 approach. In essence, this approach failed to generate a full knockout clone, possibly because of the essential role of this protein in the biology of the cell lines. Still, we generated multiple mutant HeLaP4 TRN-SR2 clones that affected HIV-1 replication to a variable extent. We pinpointed the replication deficit in these clones to a nuclear import defect, excluded indirect effects on CSPF6 or splicing, and corroborated an interface mutant (ΔV_105_ TRN-SR2) for the interaction with HIV-1 IN. Our data support a model whereby nuclear import is mediated by the interaction between HIV-1 IN and TRN-SR2 in cell lines.

## RESULTS

### A CRISPR/Cas9 knockout approach targeting *TNPO3*.

**FIG 1:**
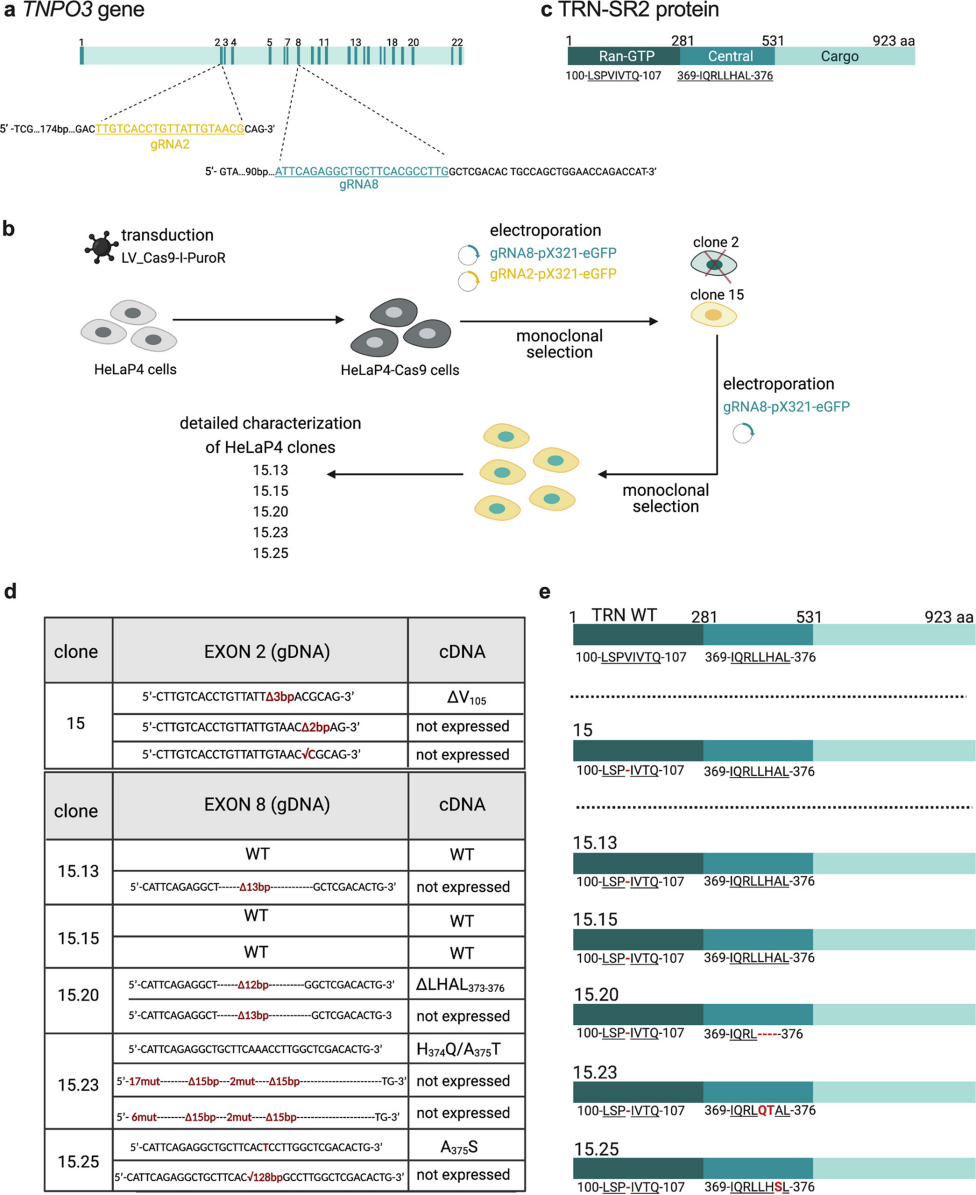
A CRISPR/Cas9 knockout approach targeting *TNPO3*. (a) Graphical representation of the *TNPO3* gene located on chromosome 7q32.1. The exons of the TRN-SR2 open reading frame (ORF) are indicated by vertical lines and numbered above the ORF. The DNA sequences of the regions in exon 2 and exon 8, which are targeted by specific guide RNAs (gRNA2 and gRNA8), are highlighted. The gRNA targeted region in each exon is underlined. (b) Schematic outline of the CRISPR/Cas9 approach. After lentiviral transduction, HeLaP4-Cas9 cells stably expressing Cas9 were selected with puromycin for 1 week. Stable polyclonal populations were subsequently electroporated with *TNPO3*-specific gRNAs (pX321-eGFP_gRNA2 and pX321-eGFP_gRNA8). eGFP-positive cells were sorted and subsequently seeded as single cells. The clones were screened based on TRN-SR2 protein levels. Clone 15 was electroporated again with pX321-eGFP_gRNA8. Finally, five clones were selected for further investigation based on their low TRN-SR2 levels. Figure created with Biorender.com. (c) Outline of the TRN-SR2 protein. Amino acid numbers (1 to 923) are indicated above the sequence together with the functional domains: (i) the Ran binding domain (amino acids 1 to 281), (ii) the central domain (amino acids 281 to 531), and (iii) the cargo binding domain (amino acids 531 to 923). CRISPR/Cas9-targeted sites are shown below the sequence, with underlined targeted amino acids. (d) Summary of the CRISPR/Cas9-induced mutagenesis on the genomic DNA (gDNA) and the transcriptional (cDNA) level. Upper panel shows clone 15, which is generated after double transfection with gRNA2 and gRNA8. The lower panel illustrates the clones which are derived from the maternal clone 15, after repeated transfection with gRNA8. WT, wild-type sequence or protein expression; Δ, deletion; bp, base pair; √, insertion; mut, mutation. (e) Outline of the wild-type TRN-SR2 protein compared to the generated mutants. Amino acid numbers (1 to 923) are indicated above the WT sequence, and CRISPR/Cas9-targeted sites are shown below the sequence, with underlined targeted amino acids.

Previously, we reported that either transient or stable depletion of TRN-SR2 from host cells by RNA interference (RNAi) reduces HIV-1 nuclear import and replication (4). Since RNAi did not completely deplete TRN-SR2, we now attempted to knock out the *TNPO3* gene using the CRISPR/Cas9 technology. Different guide RNAs (gRNAs) were designed that target different coding exons of *TNPO3* (Fig. 1a). To this end, four different plasmids encoding gRNAs targeting exons 1, 2, 3, and 8 (Table S1) were transfected together with a Cas9-expressing plasmid in 293T cells. gRNA2 and gRNA8 were selected based on their ability to reduce TRN-SR2 protein expression. In parallel, we generated HeLaP4 cells stably expressing Cas9 following LV_Cas9-I-PuroR transduction and puromycin selection. The resulting polyclonal cell line was subsequently used to electroporate both gRNA2- and gRNA8-encoding plasmids (pX321-eGFP_gRNA2 and pX321-eGFP_gRNA8; Fig. 1b). Combining two gRNA targets theoretically increases the chance of generating a protein knockout (KO) by disruption of the open reading frame (ORF). Forty-eight hours later, cells were sorted for enhanced green fluorescent protein (eGFP) using fluorescence activated cell sorting (FACS) and were allowed to expand for 1 week before seeding for monoclonal expansion into a 96-well plate. Two clones were selected (clone 2 and clone 15) with potent TRN-SR2 depletion in line with stable KD (95% and 85%, respectively; Fig. S1a) on protein levels. Single-round HIV-1 infectivity was reduced by 30 to 70% compared to that of the HeLaP4 cells transduced with short hairpin RNA (shRNA) scrambled control vector (SCR), which was still higher than the level observed in the HeLaP4 TRN-SR2 KD cells (Fig. S1b). Due to its impaired growth, we excluded clone 2 for the further studies.

We characterized the selected clone 15 on both genomic DNA (gDNA) and mRNA (cDNA) level by sequencing. Since this clone was selected from populations exposed to gRNA2 and gRNA8 (outlined in Fig. 1a), we amplified and sequenced the regions flanking both targeted sites. gRNA2 targets exon 2 of the *TNPO3* gene (amino acids 100 to 107), encoding the Ran-GTP binding domain of TRN-SR2 (outlined in Fig. 1c). gRNA8 targets exon 8 (amino acids 369 and 376), encoding the central TRN-SR2 domain (outlined in Fig. 1c). We identified and sequenced three alleles, indicating that the *TNPO3* locus is triploid in the investigated HeLaP4 cells (Fig. 1d). When analyzing the gRNA2 target region (upper part of the table in Fig. 1d), we observed that each CRISPR/Cas9-treated cell line contains three alleles: (i) one with a 3-base-pair (bp) deletion (Δ3bp), (ii) one with a 2-bp deletion (Δ2bp), and (iii) one with an inserted cytosine (√C). Of these three alleles, only the first (carrying an in-frame Δ3bp) was detected when the respective cDNA amplicon was sequenced, suggesting that the other mRNA transcripts carrying out-of-frame indels are most probably degraded following nonsense-mediated mRNA decay (NMD). The former transcript is predicted to translate into TRN-SR2 protein with a deletion of Val at position 105 in TRN-SR2. Therefore, next to the lower mRNA expression levels due to two out-of-frame alleles, Val 105 is deleted in the N terminus of the translated protein in each of the selected clones (ΔV_105_ TRN-SR2). Analysis of the gRNA8 target region for clone 15 did not indicate any alteration compared to the wild-type *TNPO3* (*TNPO3^WT^*) sequence, at both genomic DNA level and cDNA level.

In an effort to further reduce the TRN-SR2 protein levels, we repeated the electroporation and sorting procedure on clone 15 with the pX321-eGFP_gRNA8 (see outline in Fig. 1b). Five HeLaP4 clones derived from the maternal clone 15 were selected based on the TRN-SR2 protein expression level relative to that of HeLaP4 TRN-SR2 KD (Fig. S1c and d): 15.13, 15.15, 15.20, 15.23, and 15.25.

### Characterization of the selected clones on gDNA and mRNA level reveals different TRN-SR2 mutant clones.

The five selected clones were further characterized on both the gDNA and the mRNA (cDNA) level by sequencing. All the clones genetically resembled the maternal clone 15 at the gRNA2 target region as described (ΔV_105_ TRN-SR2). When analyzing the gRNA8 target region (lower part of the table in Fig. 1d), we observed three distinct alleles in clone 15.23 and two alleles in all other clones. clone 15.13 contained one *TNPO3*^WT^ allele and one allele with a 13-bp deletion (Δ13 bp) that theoretically results in a frameshift. cDNA analysis for the latter region showed only *TNPO3*^WT^, suggesting NMD for the mutated mRNA. In clone 15.15, both *TNPO3* alleles were WT and we detected WT mRNA expression. In clone 15.20, we detected two mutated *TNPO3* alleles: one with a 13-bp deletion (Δ13 bp) with no mRNA expression, which was not detected at cDNA level either, and one with a 12-bp (Δ12) in-frame deletion, which yielded the corresponding mRNA, resulting in deletion of LHAL amino acids (ΔLHAL; positions 373 to 376 in TRN-SR2). Clone 15.23 had multiple deletions in two of its *TNPO3* alleles, and these could not be detected in the cDNA analysis, suggesting NMD, whereas only the third one, with a CG-to-AA exchange (CG→AA) at the gDNA level, was also detected in the cDNA sequencing, resulting in a two-amino-acid exchange in TRN-SR2 (H_374_A→Q_374_T; HA→QT). In clone 15.25, the only *TNPO3* allele which was transcribed to mRNA presented with a G-to-T point mutation (G→T), changing the Ala 375 in TRN-SR2 to Ser (A_375_S). The other allele of clone 15.25 showed a 128-bp deletion (Δ128 bp) and was not detected in the cDNA analysis (NMD). Altogether, sequencing revealed that all clones showed mRNA levels lower than those of wild-type cells, most probably due to NMD of the transcripts carrying out-of-frame indels, since these could not be picked up in our cDNA analysis. All clones presented with indels: (i) a Δ3 bp at the gRNA2 binding site, resulting in deletion of Val at position 105 in TRN-SR2 (ΔV_105_ TRN-SR2), and (ii) deletions or substitutions, leading to mutated TRN-SR2 in the gRNA8 target region (ΔLHAL_373–376_ TRN-SR2; H_374_Q/A_375_T TRN-SR2; A_375_S TRN-SR2). Interestingly, only clone 15.15 genetically resembles the maternal clone 15 and therefore represents an ideal control, solely carrying the gRNA2 ΔV_105_ TRN-SR2 edit. The schematic representation of the resulting TRN-SR2 proteins is outlined in Figure 1e. Although we did not obtain a full knockout of *TNPO3* in these HeLaP4 cells, we reasoned that the various mutations and/or deletions in TRN-SR2 may represent interface mutants, with HIV-1 IN affecting HIV-1 replication and nuclear import. Therefore, we decided to characterize these clones in detail.

### TRN-SR2 mutant clones of variable expression show impaired single-round HIV-1 transduction.

In a more in-depth analysis, we next determined TRN-SR2 mRNA and protein levels in the five selected clones to quantify KD efficiency. As shown in Figure S1c, TRN-SR2 mRNA levels were reduced in all the clones by at least 50% compared to those of the HeLaP4 WT_SCR_ control, which was generated as described previously (13). All HeLaP4 clones displayed decreased TRN-SR2 protein levels in cell lysates compared to those in the WT_SCR_ control. We observed 15% (TRN-SR2 KD and 15.20), 30% (15.13 and 15.23), and 35% (15.15 and 15.25) TRN-SR2 protein expression relative to that of the WT_SCR_ control (Fig. S1d).

When these clones were challenged with single-round HIV-1, infectivity was reduced by 50 to 75% in the clones compared to that in the HeLaP4 WT_SCR_ control, except for the clone 15.23 (Fig. S1e). Single-round transduction was most severely affected for clone 15.20, with a reduction of 75% compared to that of the WT_SCR_ control (*P* < 0.0001; Fig. S1e). For further experiments, we excluded clone 15.13, due to its poor growth in culture, and clone 15.23, for the lack of effect on HIV-1 single-round transduction (Fig. S1e).

### Replication deficit during single-round HIV-1 transduction and multiple-round HIV-1 replication in TRN-SR2 mutant clones is rescued by overexpression of wild-type TRN-SR2.

**FIG 2:**
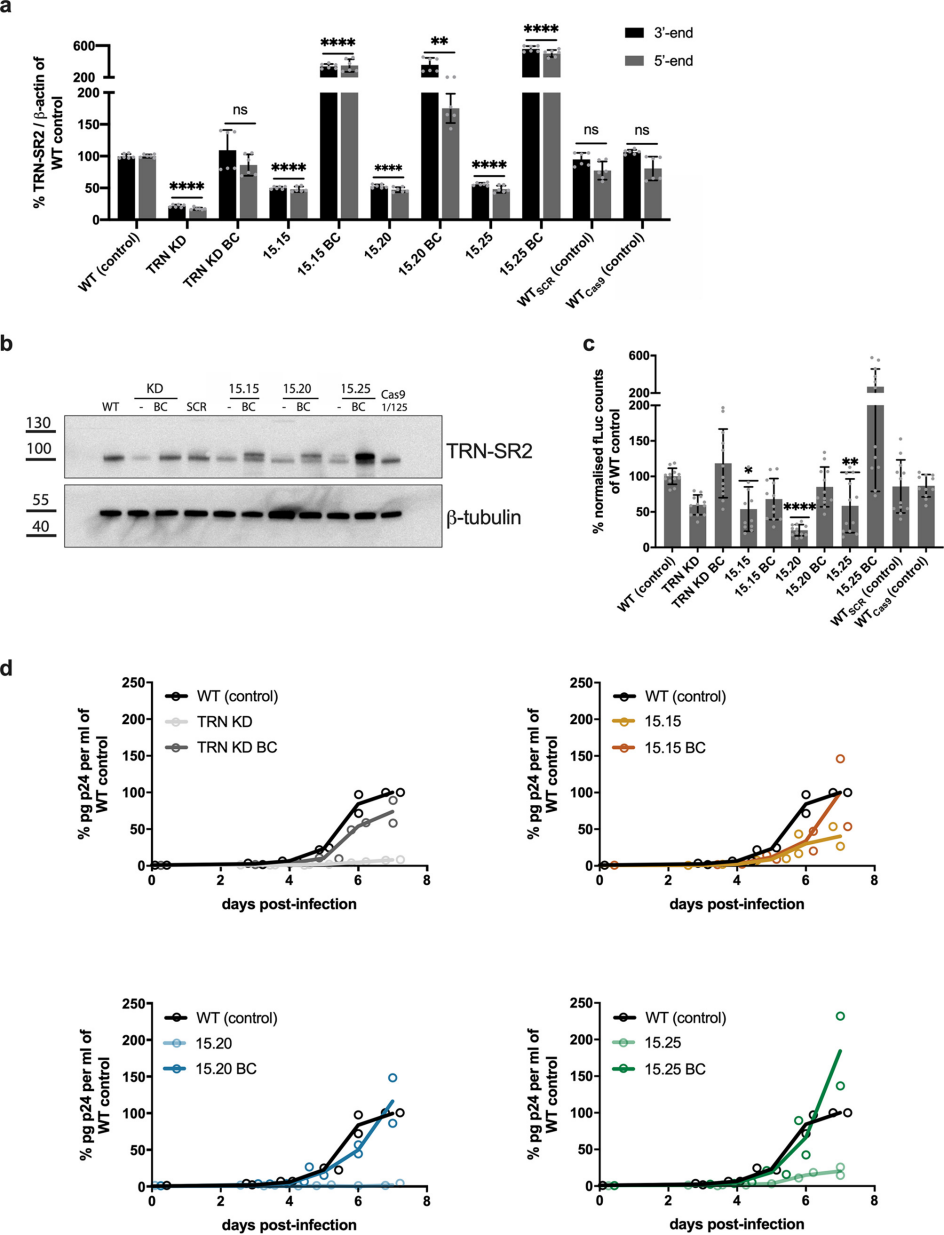
Replication deficit during single-round HIV-1 transduction and multiple-round HIV-1 replication in TRN-SR2 mutant clones is rescued by overexpression of wild-type TRN-SR2. (a) The levels of TRN-SR2 mRNA determined by reverse transcriptase quantitative PCR (RT-qPCR), using primers specific for either the 5′ or the 3′end of TRN-SR2 mRNA (for sequences see Materials and Methods). Mean and standard deviation of two independent experiments, each performed in triplicate, are presented. A repeated measures two-factor analysis of variance (ANOVA) was used to compare the TRN-SR2 mRNA expression in each condition (TRN-SR2 KD, HeLaP4 clones, and back-complemented cells) to that in HeLaP4 WT control: ns, not significant; **, *P* < 0.01; ****, *P* < 0.0001. (b) TRN-SR2 protein levels were estimated by Western blotting in control cells (HeLaP4 WT, WT_SCR_, and WT_Cas9_), HeLaP4 TRN-SR2 KD, and HeLaP4 clones (15.15, 15.20, and 15.25) and back-complemented clones, using a TRN-SR2 specific antibody and a beta-tubulin antibody as the loading control. For the back-complemented clones, a slight difference in protein mobility was observed, as indicated by the upper and lower band of TRN-SR2. Western blot signals were quantified with densitometry (Fiji). (c) Control (HeLaP4 WT, WT_SCR_, and WT_Cas9_), HeLaP4 TRN-SR2 KD, HeLaP4 clones (15.15, 15.20, and 15.25), and back-complemented clones were infected with single-round HIV-1 expressing firefly luciferase. Luciferase activity was measured 72 h posttransduction and normalized for the total protein content. Data from three different virus dilutions (1/1, 1/5, and 1/25) are represented as relative infectivity compared to infection in HeLaP4 WT control. Mean and standard deviation of two independent experiments, each performed in duplicate, are presented. A Kruskal-Wallis test was used to test for statistical significance compared to HeLaP4 WT control: *, *P* < 0.05; **, *P* < 0.01; ****, *P* < 0.0001. (d) Control (HeLaP4 WT), HeLaP4 TRN-SR2 KD, HeLaP4 clones (15.15, 15.20, and 15.25), and back-complemented clones were infected with replication-competent HIV-1 NL4.3. Replication was monitored by sampling p24 at regular intervals, starting 3 days after infection. The mean p24 value is shown from duplicate data points (displayed as circles) from one of two representative experiments.

To exclude clonal or off-target effects, we back-complemented the HeLaP4 TRN-SR2 KD cells and the respective HeLaP4 clones with stably expressed ectopic TRN-SR2^WT^ driven by a potent cytomegalovirus (CMV) promoter. The construct was resistant to the shRNA, used to generate TRN-SR2 KD cells. Next to the HeLaP4 WT control, the HeLaP4 WT_SCR_ and the HeLaP4 WT_Cas9_ expressing stable Cas9 were included as controls. As shown in Figure 2a, overexpression of TRN-SR2^WT^ increased total TRN-SR2 mRNA levels 3- to 6-fold in the HeLaP4 complemented clones compared to those in the HeLaP4 WT control. In contrast, back-complementation of HeLaP4 TRN-SR2 KD cells increased overall TRN-SR2 mRNA to HeLaP4 WT-like levels. When the protein expression was analyzed by Western blotting, the clones showed 30% (15.20) and 50% (15.15 and 15.25) of the TRN-SR2 expression of the HeLaP4 WT control (Fig. 2b). Notably, we did not reach the KD level obtained by shRNA-mediated TRN-SR2 KD (20%). Overexpression of TRN-SR2^WT^ increased overall protein levels almost 3-fold in clone 15.15 and 15.20 and 5-fold in clone 15.25 relative to those of the HeLaP4 WT control.

**FIG 3:**
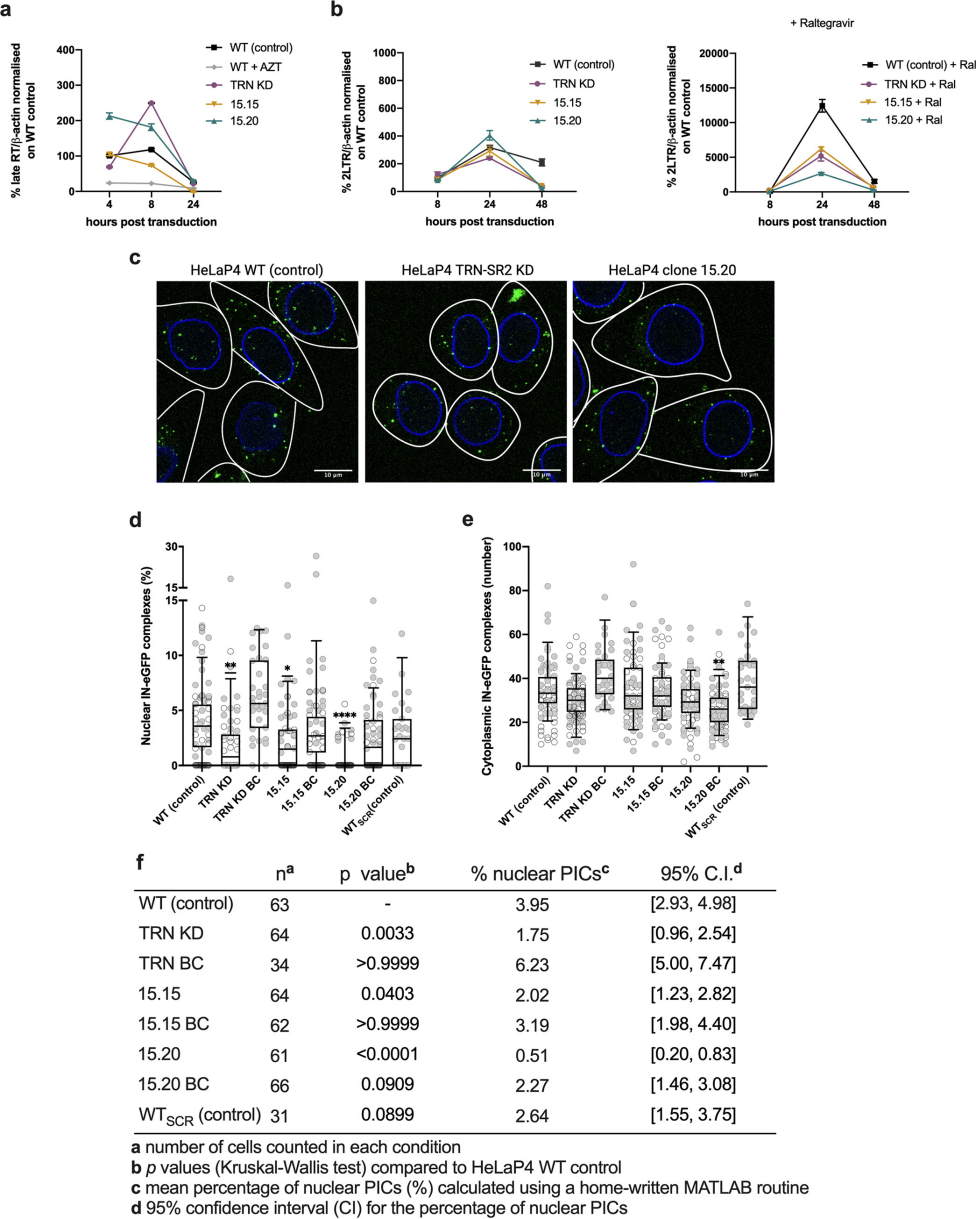
HIV-1 replication in TRN-SR2 mutant clones is blocked at the level of nuclear import. (a and b) Analysis by qPCR of viral cDNA synthesis (late reverse transcripts) and nuclear import (2-LTR circles). Cells were transduced with HIV-1 NL4.3 and harvested at distinct time points after transduction. AZT (RT inhibitor) and raltegravir (Ral, integration inhibitor) were included as controls, respectively. (a) Late reverse transcripts, means ± SD are shown from one of two representative experiments, performed in duplicate. A Kruskal-Wallis test was used to test for statistical significance compared to HeLaP4 WT control (*, *P* < 0.05 for WT plus AZT, at 4 h and 8 h posttransduction). (b) Number of 2-LTR circles, means ± SD are shown from one of two representative experiments, performed in duplicate. A Kruskal-Wallis test was used to test for statistical significance compared to HeLaP4 WT control (**, *P* < 0.01 for 15.20 at 24 and 48 h posttransduction). (c) The PIC nuclear import assay was performed as described previously (21). Control (HeLaP4 WT and WT_SCR_), HeLaP4 TRN-SR2 KD, HeLaP4 clones (15.15, 15.20), and back-complemented cells were transduced with replication-deficient IN-eGFP HIV-1. Cells were fixed 6 h posttransduction and IN-eGFP-labeled PICs were detected by confocal microscopy. Three representative images are shown: control (HeLaP4 WT), HeLaP4 TRN-SR2 KD, and clone 15.20. An automated linear adjustment of brightness and contrast was applied on the entire images using Fiji for better visualization of the PICs (pseudocolored green). (d and e) The percentage of nuclear IN-eGFP complexes (ratio nuclear/total PICs) (d) and the number of cytoplasmic IN-eGFP complexes (e) were analyzed with an in-house MATLAB routine. Box-and-whisker plots (5 to 95 percentile) overlapping individual points are shown from two independent experiments, except for HeLaP4 WT_SCR_ and TRN KD BC. (f) PIC assay statistics. A Kruskal-Wallis test was done to test for statistical significance compared to HeLaP4 WT control: *, *P* < 0.05; **, *P* < 0.01; ****, *P* < 0.0001.

We next transduced the TRN-SR2 KD cells, HeLaP4 clones, and back-complemented cells with single-round HIV-1. TRN-SR2^WT^ overexpression restored single-round HIV-1 infectivity in HeLaP4 TRN-SR2 KD cells to the levels observed in HeLaP4 WT control. In clones 15.15 and 15.20, back-complementation partially restored single-round HIV-1 infectivity (Fig. 2c), despite 3- to 6-fold overexpression of TRN-SR2 (Fig. 2a). In clone 15.25, single-round transduction increased 2-fold compared to that of the HeLaP4 WT control (Fig. 3a). Next, we evaluated the susceptibility of the TRN-SR2 clones to support multiple-round HIV-1 replication (Fig. 2d). We infected the HeLaP4 TRN-SR2 KD, HeLaP4 clones, and back-complemented cells with replication-competent HIV-1 NL4.3 and monitored replication by sampling p24 at regular intervals. The strongest effect was seen for clone 15.20, which did not permit HIV-1 breakthrough even after 7 days. An intermediate effect was observed for clone 15.15 and 15.25, where HIV-1 replication was reduced to 40% or 20% of the levels observed in the HeLaP4 WT control, respectively. Overexpression of TRN-SR2^WT^ in clone 15.15 and 15.20 restored multiple-round HIV-1 replication, but HIV-1 breakthrough was still delayed compared to that of the HeLaP4 WT control. A full rescue of HIV-1 replication was obtained after overexpression of TRN-SR2^WT^ in clone 15.25, which supported more virus replication than the HeLaP4 WT control (Fig. 3b). Additionally, growth curve analysis revealed no difference in cell proliferation between the HeLaP4 WT control, HeLaP4 TRN-SR2 KD, HeLaP4 clones, and back-complemented HeLaP4 cells (Fig. S2). To keep the study focused while retaining the ability to distinguish between effects linked to mutagenesis in exon 2 alone, or combined with mutagenesis in exon 8, we decided to continue with clone 15.15 (ΔV_105_ exon 2; WT exon 8) and 15.20 (ΔV_105_ exon 2; ΔLHAL_373–376_ exon 8) and exclude clone 15.25 (ΔV_105_ exon 2; A_375_S exon 8) for further analysis. Additionally, we determined the TRN-SR2 expression levels with immunocytochemistry (Fig. S3). The most pronounced reduction in TRN-SR2 expression was obtained in the HeLaP4 TRN-SR2 KD. Clone 15.15 and 15.20 both carry alleles that do not support mRNA expression and alleles that generate mutant TRN-SR2 resulting in intermediate expression levels of TRN-SR2. Overall, overexpression of wild-type TRN-SR2 increased protein levels in back-complemented cell lines to WT-like levels.

### HIV-1 replication in TRN-SR2 mutant clones is blocked at the level of nuclear import.

To pinpoint the replication step affected by the CRISPR/Cas9-induced mutants, we monitored formation of viral DNA intermediates by quantitative PCR (qPCR) analysis for late reverse transcripts, 2-long-terminal-repeat (2-LTR) circles, and integrated proviruses. HeLaP4 WT control cells, TRN-SR2 KD cells, and HeLaP4 clones were transduced with replication-deficient HIV-1 NL4.3 and late reverse transcripts were measured by qPCR at 4, 8 and 24 h posttransduction (late reverse transcripts [RT], Fig. 3a). Azidothymidine (AZT) was included as chain terminator to inhibit reverse transcription. The number of late reverse transcripts was not significantly altered between different conditions, except for the control condition supplemented with AZT, in which reverse transcription was blocked (*P* < 0.05, at 4 and 8 h posttransduction). It has been described previously that TRN-SR2 depletion results in a reduction of 2-LTR circles during HIV-1 infection (4, 13, 33), and hence we measured the formation of 2-LTR circles as an indirect measure for nuclear import (2). To this end, the cells were transduced with replication-deficient HIV-1 NL4.3 and 2-LTR circles were measured by qPCR at 8, 24 and 48 h posttransduction (2-LTR, Fig. 3b). In agreement with previous studies (34), inhibition of integration by addition of Ral increased 2-LTR circles more than 10-fold in HelaP4 WT control cells (Fig. 3b). The increase in 2-LTR circles in HeLaP4 TRN-SR2 KD cells and clone 15.15 and 15.20 after addition of Ral did not reach levels obtained in HeLaP4 WT control cells (*P* < 0.01 for 15.20 at 24 and 48 h posttransduction), suggesting a nuclear import defect. Back-complementation increased 2-LTR circles 2-fold in HeLaP4 TRN-SR2 KD cells (Fig. S4a and S4b), whereas back-complementation of the HeLaP4 clones did not increase 2-LTR circles. Finally, the number of integrated proviruses was determined by measuring the firefly luciferase (fLuc) coding region by qPCR 10 days posttransduction to exclude nonintegrated DNA and decreased by 70% (TRN-SR2 KD and 15.20) and 40% (15.15, Fig. S1f); this defect was partially rescued after back-complementation with TRN-SR2^WT^ (Fig. S1f).

To analyze the HIV-1 nuclear import defect in more detail and provide orthogonal data, we performed a PIC nuclear import assay. Six hours after transduction with IN-eGFP-labeled viruses, fluorescently labeled PICs were visualized using confocal microscopy and analyzed using an in-house MATLAB routine (33). Three representative images (HeLaP4 WT, HeLaP4 TRN-SR2 KD, and clone 15.20) are shown in Figure 3c. The subcellular localization of the IN-eGFP complexes (pseudocolored green in the images) was evaluated based on lamin staining (pseudocolored blue in the images), and the IN-eGFP complexes were assigned to the cytoplasmic or the nuclear compartment (Fig. 3c). The ratio of nuclear PICs over the total number of PICs (nuclear IN-eGFP complexes percentage) was calculated as a measure of nuclear import. Compared to that in the HeLaP4 WT control, the ratio of nuclear PICs was reduced 2-fold in HeLaP4 TRN-SR2 KD cells and in clone 15.15 and almost 8-fold in clone 15.20 (Fig. 3d; *P* < 0.01, *P* < 0.05, and *P* < 0.0001 for TRN-SR2 KD, 15.15, and 15.20, respectively). Reduced infectivity was excluded since no decrease in cytoplasmic IN-eGFP complexes was detected in the respective cell lines compared to those in the HeLaP4 WT control (Fig. 3e). After back-complementation with TRN-SR2^WT^, nuclear import of IN-eGFP complexes increased 3-fold, almost 2-fold, and 4-fold in TRN-SR2 KD cells, clone 15.15, and 15.20, respectively. Altogether, based on both qPCR and PIC-import data, CRISPR/Cas9-induced TRN-SR2 mutations appear to result in a block in nuclear import of HIV-1.

### CRISPR/Cas9-induced mutagenesis generates a TRN-SR2/IN interface mutant.

**FIG 4:**
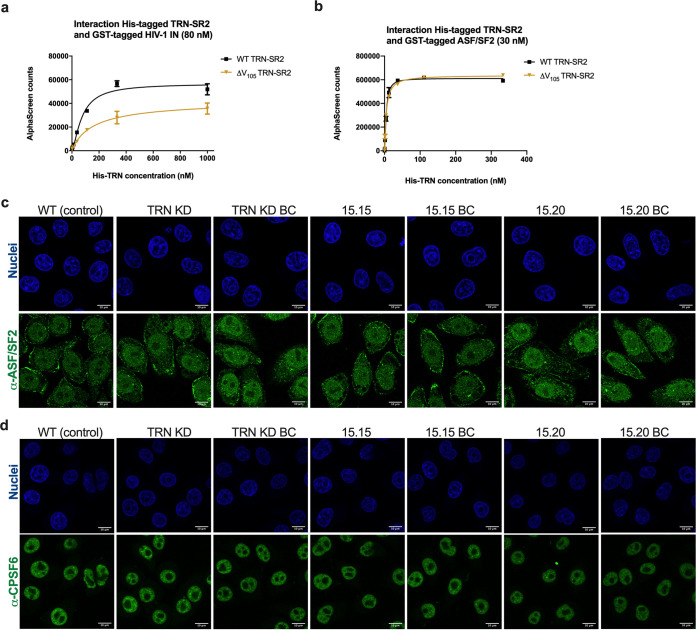
CRISPR/Cas9-induced mutagenesis generates a TRN-SR2/IN interface mutant but does not affect nuclear import of SR proteins. AlphaScreen-based analysis of the interaction between (a) His_6_-tagged WT TRN-SR2 or His_6_-tagged ΔV_105_ TRN-SR2 (corresponding to TRN-SR2 protein expressed in clone 15.15) with GST-tagged integrase (IN). TRN-SR2 protein was titrated over a fixed concentration of IN (80 nM). (b) His_6_-tagged WT TRN-SR2 or His_6_-tagged ΔV_105_ TRN-SR2 with GST-tagged ASF/SF2. TRN-SR2 protein was titrated over a fixed concentration of ASF/SF2 (30 nM). Mean and standard deviation are shown from one of two representative experiments, performed in duplicate. (c and d) Control (HeLaP4 WT), HeLaP4 TRN-SR2 KD, HeLaP4 clones, and back-complemented cells were fixed and immunostained with monoclonal antibodies against (c) ASF/SF2 (an automated linear adjustment of brightness and contrast was applied on the entire images using Fiji) and (d) CPSF6. Secondary antibody was conjugated to Alexa 488 (pseudocolored green) while the nucleus was stained with DAPI (pseudocolored blue). Scale bars represent 10 μm. One representative experiment, including the parallel analysis of all cell lines, of two experiments is shown.

According to one of the models, TRN-SR2 acts as a cellular cofactor of HIV-1 nuclear import through a direct interaction with the viral IN (4, 8, 13, 28). To investigate the underlying mechanism responsible for the observed nuclear import defect in the selected HeLaP4 clones, we determined binding affinity of the TRN-SR2 mutant proteins for the HIV-1 IN and ASF/SF2, a natural cargo of TRN-SR2. We were able to express and purify recombinant WT TRN-SR2 and ΔV_105_ TRN-SR2 corresponding to the protein expressed in clone 15.15. In turn, for recombinant ΔLHAL_373–376_ TRN-SR2 and ΔV_105_ ΔLHAL_373–376_ TRN-SR2 (corresponding to protein expressed in clone 15.20), soluble protein could not be purified to sufficient yields. The signal of the AlphaScreen protein-protein interaction assay for the interaction between HIV-1 IN and ΔV_105_ TRN-SR2 was 2-fold lower than that for the interaction between HIV-1 IN and WT TRN-SR2 (Fig. 4a), consistent with a 2-fold reduction in HIV-1 infectivity (Fig. 2c and d) and a 2-fold inhibition of nuclear import (Fig. 3d). In contrast, the affinity of ΔV_105_ TRN-SR2 for ASF/SF2 was comparable to that of WT protein (Fig. 4b), indicating that CRISPR/Cas9-induced mutagenesis in TRN-SR2 in clone 15.15 did not affect the interaction with SR-rich cargo proteins. These protein-protein interaction data were reproduced with alternative protein tags (Fig. S5).

### Cellular functions of TRN-SR2 are not affected in the TRN-SR2 mutant clones.

**FIG 5:**
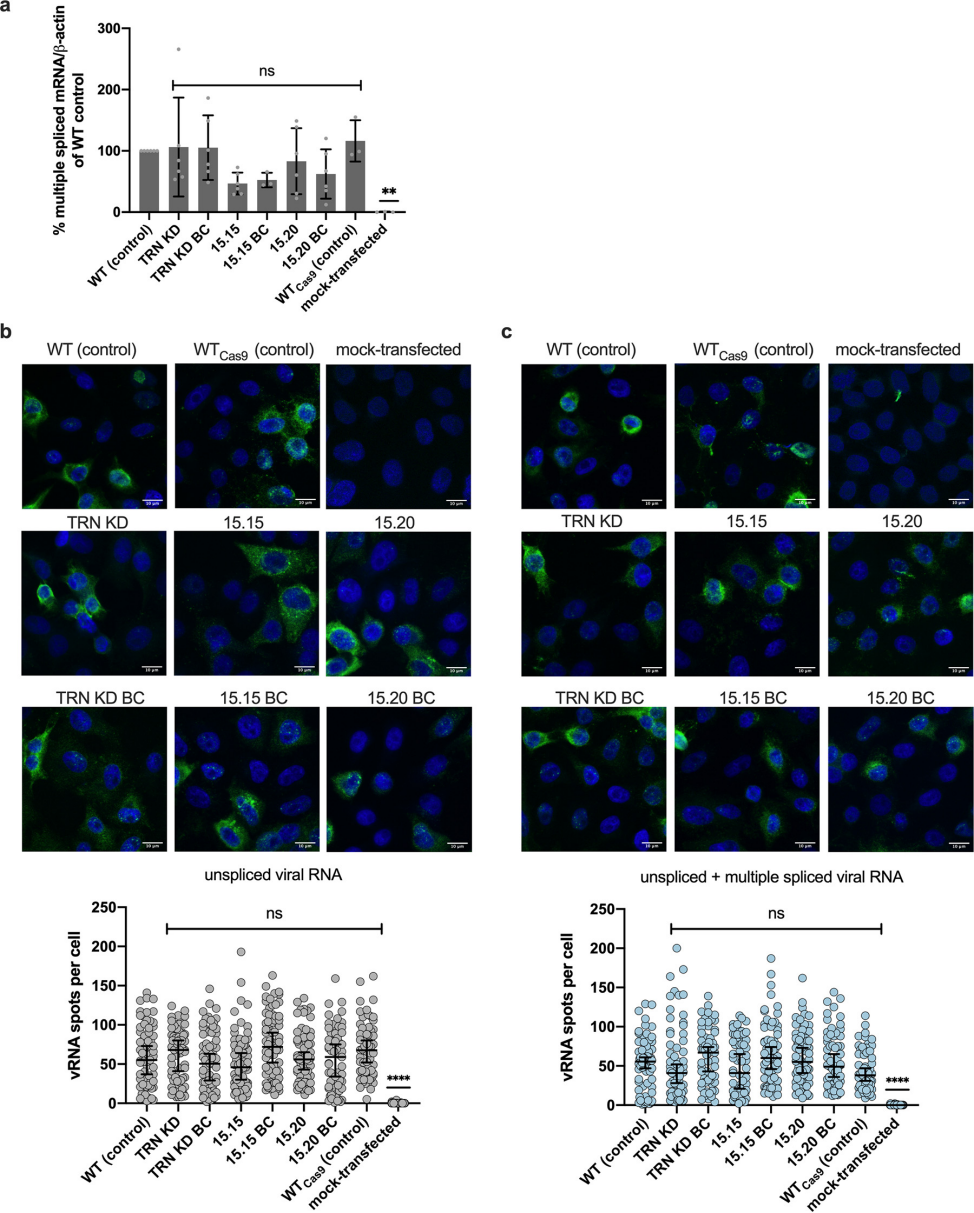
Viral mRNA splicing is not altered in TRN-SR2 mutant clones. (a) Multiple-spliced viral mRNA as determined by RT-qPCR in control (HeLaP4 WT and WT_Cas9_), HeLaP4 TRN-SR2 KD, HeLaP4 clones, back-complemented cells, and mock-transfected cells (negative control) as a functional readout of TRN-SR2 activity. Mean and standard deviation are shown from six independent experiments, except for WT_Cas9_ (*n* = 3), each performed in triplicate. A Kruskal-Wallis test was done to test for statistical significance compared to HeLaP4 WT control: ns, not significant; **, *P* < 0.01. (b) Unspliced viral mRNA and (c) total viral mRNA as determined by RNAscope analysis in control (HeLaP4 WT and WT_Cas9_), HeLaP4 TRN-SR2 KD, HeLaP4 clones, back-complemented cells and mock-transfected cells (negative control). The following numbers of cells were imaged to determine unspliced vRNA and total vRNA, respectively: HeLaP4 WT (control), *n* = 69 and *n* = 72; TRN-SR2 KD, *n* = 73 and *n* = 65; TRN-SR2 KD BC, *n* = 74 and *n* = 63, 15.15, *n* = 76 and *n* = 65; 15.15 BC, *n* = 74 and *n* = 67; 15.20, *n* = 68 and *n* = 76; 15.20 BC, *n* = 69 and *n* = 65; HeLaP4 WT_Cas9_ (control), *n* = 66 and *n* = 63; and mock-transfected cells, *n* = 50 and *n* =52. One representative experiment, including the parallel analysis of all cell lines, of two experiments is shown. The number of viral RNA spots per cell was analyzed with an in-house MATLAB routine. Medians with 95% confidence intervals are shown for overlapping individual points from one of two representative experiments. A Kruskal-Wallis test was done to test for statistical significance: ns, not significant; ****, *P* < 0.0001.

TRN-SR2 imports mRNA splicing factors such as ASF/SF2 and CPSF6 into the nucleus (29). According to some (7, 29), but not all studies (23, 35), both CPSF6 and ASF/SF2 can accumulate in the cytoplasm following TRN-SR2 KD. Therefore, we analyzed the cellular distribution of ASF/SF2 and CPSF6 in the HeLaP4 WT control cells, TRN-SR2 KD cells, HeLaP4 clones, and back-complemented cells. The cellular distribution of CPSF6 and ASF/SF2 did not change in TRN-SR2 KD or HeLaP4 clones in comparison to that in the HeLaP4 WT control (Fig. 4c and d). ASF/SF2 is an essential splicing factor involved in pre-mRNA splicing of cellular but also HIV-1 RNA (36). Since HIV-1 is highly dependent on the splicing machinery of the cell, perturbing levels of ASF/SF2 may inhibit HIV-1 replication (37). To further confirm that the observed inhibition of HIV-1 replication in the HeLaP4 clones is not due to an altered cellular distribution of ASF/SF2, the cells were transfected with NL4.3-based plasmid and the multiple-spliced HIV-1 mRNA levels were determined with qPCR and RNAscope technology. As outlined in Figure 5, there was no significant change in multiple-spliced mRNA levels in the HeLaP4 clones and back-complemented clones compared to that in control cells as determined with qPCR (Fig. 5a). In addition, we quantified the level of multiple-spliced viral RNA (vRNA) using RNAscope technology. We used a first RNA probe to detect unspliced and multiple-spliced vRNA and a second probe specific for unspliced vRNA, allowing us to indirectly quantify changes in multiple-spliced vRNA (Fig. 5b and c). No significant changes in total vRNA (unspliced and multiple-spliced viral RNA) and unspliced vRNA were observed in the HeLaP4 clones compared to those in the HeLaP4 WT control, except for the mock-transfected cells (negative control, *P* < 0.0001; Fig. 5b and c), indicating that splicing of viral RNA is not altered in the CRISPR/Cas9-treated and back-complemented cells.

### Infectivity of VSV-g pseudotyped, CPSF6-independent N74D CA HIV-1 is impaired in TRN-SR2 mutant clones.

**FIG 6:**
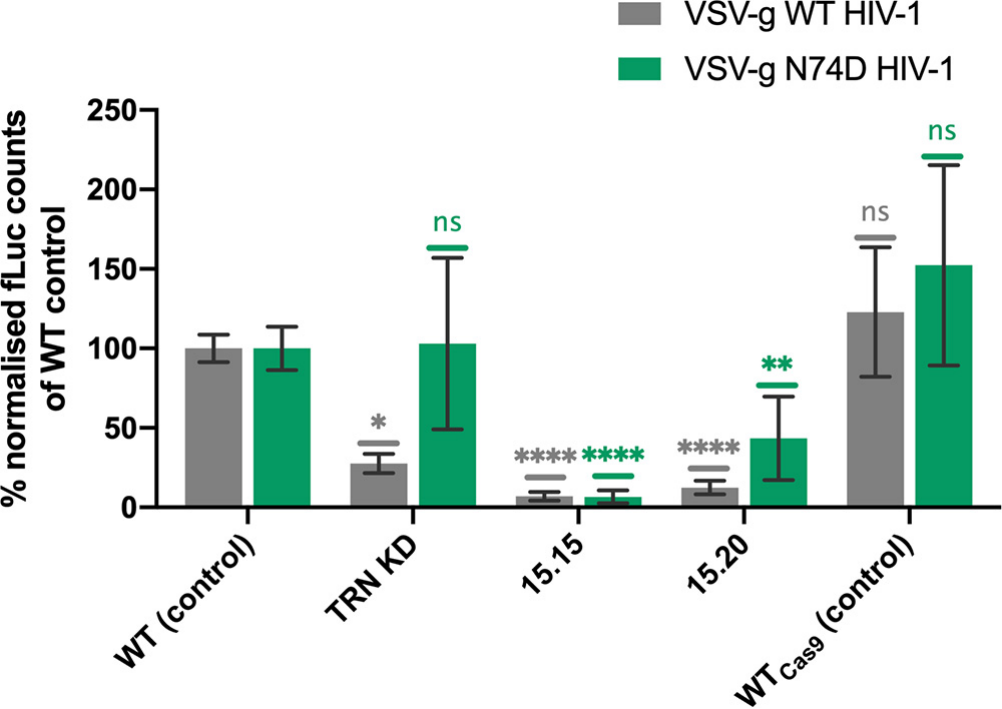
Infectivity of VSV-g pseudotyped, CPSF6-independent N74D CA HIV-1 is impaired in TRN-SR2 mutant clones. Control (HeLaP4 WT, WT_Cas9_), HeLaP4 TRN-SR2 KD, and HeLaP4 clones were transduced with wild-type or N74D CA single-round HIV-1 expressing firefly luciferase reporter pseudotyped with VSV-g. Luciferase activity was measured 72 h posttransduction and normalized for the total protein content. Data from four different virus dilutions (1/300, 1/900, 1/2,700, and 1/8,100) are represented as relative infectivity compared to that of HeLaP4 WT control. Mean and standard deviation of two independent experiments, each performed in duplicate, are presented. A Kruskal-Wallis test was used to test for statistical significance compared to HeLaP4 WT control: ns, not significant; *, *P* < 0.05; **, *P* < 0.01; ****, *P* < 0.0001.

In single-round experiments, vesicular stomatitis virus G (VSV-g) pseudotyped HIV-1 carrying the N74D CA mutation transduces cells independently of TRN-SR2 (11, 13, 16, 38–40). Since the N74D mutant virus was additionally shown to replicate independently of NUP153 and NUP358, it may use an alternative import pathway (11, 38). N74D virus does not interact with CPSF6 and is often used in experiments to evaluate the effect of CPSF6. We investigated if replication of the N74D HIV-1 was affected by the CRISPR/Cas9-induced TRN-SR2 mutations. Therefore, we compared single-round transduction efficiency between VSV-g pseudotyped WT and N74D HIV-1 in HeLaP4 control, HeLaP4 TRN-SR2 KD, and HeLaP4 clones. Consistently with previous reports (11, 13, 16, 38), single-round N74D HIV-1, but not WT HIV-1, was insensitive to TRN-SR2 knockdown. Interestingly, infectivity of N74D HIV-1 was reduced 15-fold in clone 15.15 and 2-fold in clone 15.20 compared to that in HeLaP4 WT control (Fig. 6). In conclusion, these data show that infectivity of the N74D HIV-1 in clone 15.15 and 15.20 remains dependent on TRN-SR2, excluding a major role of CPSF6 in the observed phenotype.

## DISCUSSION

A CRISPR/Cas9 gene knockout approach targeting exon 2 and exon 8 of the *TNPO3* gene failed to generate complete knockout clones. We assume that full knockouts were generated with our approach but that these did not generate viable clones, indicating that TRN-SR2 is an essential protein, at least in these HeLaP4 cells. Although a *TNPO3* knockout in primary cells has been reported, this occurred with only a 40 to 70% editing efficiency depending on the donor (41). The triploidy of the HeLaP4 cell line may have contributed to the technical challenge. Still, our CRISPR/Cas9 approach yielded indel mutants of *TNPO3*. Genome sequencing of *TNPO3* revealed that CRISPR/Cas9 targeting induced out-of-frame alleles in both exon 2 and exon 8, resulting in reduced mRNA expression levels, while the remaining alleles showed small in-frame deletions or substitutions resulting in mutant TRN-SR2 clones. The resulting clones were fully viable but failed to support HIV-1 replication. The block in HIV-1 replication was subsequently pinpointed to the nuclear import step, and the TRN-SR2 mutant in clone 15.15 was characterized as encoded by a single *TNPO3* allele producing ΔV_105_ TRN-SR2. The corresponding recombinant ΔV_105_ TRN-SR2 was impaired 2-fold for interaction with HIV-1 IN and thus represents an interface mutant. Our results indicate that not only the extent of TRN-SR2 depletion but also the induced mutations in TRN-SR2 highly affected HIV-1 infectivity. Although characterized by comparable levels of residual TRN-SR2 expression, single-round HIV-1 transduction was severely hampered in clone 15.20 but not in 15.23. In theory, the additional mutations in clone 15.23 (H_374_A→Q_374_T) might rescue the loss of interaction with IN. However, clone 15.23 was not further investigated and therefore no experimental data are available that support this hypothesis.

The selected clones displayed reduced TRN-SR2 mRNA and protein levels compared to those of the HeLaP4 WT control cells (Fig. 2a and b). We observed comparable mRNA levels using two different primer sets, annealing to either the 5′ end or the 3′ end of TRN-SR2 mRNA (Fig. 2a). The most pronounced reduction in TRN-SR2 mRNA and protein expression was observed for clone 15.20, although we did not reach the extent of protein depletion observed after shRNA-mediated KD. Intermediate TRN-SR2 mRNA and protein expression were observed for clone 15.15 and 15.25 (Fig. 2a and b). Clone 15.20 was most strongly impaired for single- and multiple-round HIV-1 replication and HIV-1 nuclear import (Fig. 2c and d and Fig. 3d). Back-complementation of HeLaP4 TRN-SR2 KD cells and HeLaP4 clones restored TRN-SR2 mRNA and protein levels (Fig. 2a and b) and at least partially rescued single- and multiple-round viral replication (Fig. 2c and d), corroborating a TRN-SR2-specific effect while excluding clonal and off-target effects. The WT_Cas9_ control excluded an indirect effect of stable Cas9 expression. Using the PIC nuclear import assay, we showed that back-complementation rescued nuclear import entirely in TRN-SR2 KD cells and at least partially in our CRISPR/Cas9-induced clones (Fig. 3d). Yet, the number of 2-LTR circles, a surrogate marker for nuclear import, was only partially rescued in HeLaP4 TRN-SR2 KD cells and not in HeLaP4 clones upon back-complementation (Fig. S4). Of note, measurement of nuclear import via 2-LTR circles has been underestimated and even misinterpreted in the past (7), highlighting the importance of imaging-based PIC nuclear import assays to directly follow nuclear import in infected cells. Furthermore, back-complementation of TRN-SR2 depleted cells may depend on regulatory mechanisms other than rescue of CRISPR/Cas9-induced mutants. Expression of mutant TRN-SR2 may also interfere with the function of wild-type protein, resulting in a dominant negative effect. Similar findings were described in a study that analyzed HIV-1 infection in cells from limb girdle muscular dystrophy 1F (LGMD1F) patients (42). Those patients are characterized by codominant expression of wild-type and mutant TRN-SR2. Despite the presence of 50% of the wild-type TRN-SR2, virus replication was reduced 18-fold in LGMD1F cells compared to that in control cells.

**FIG 7:**
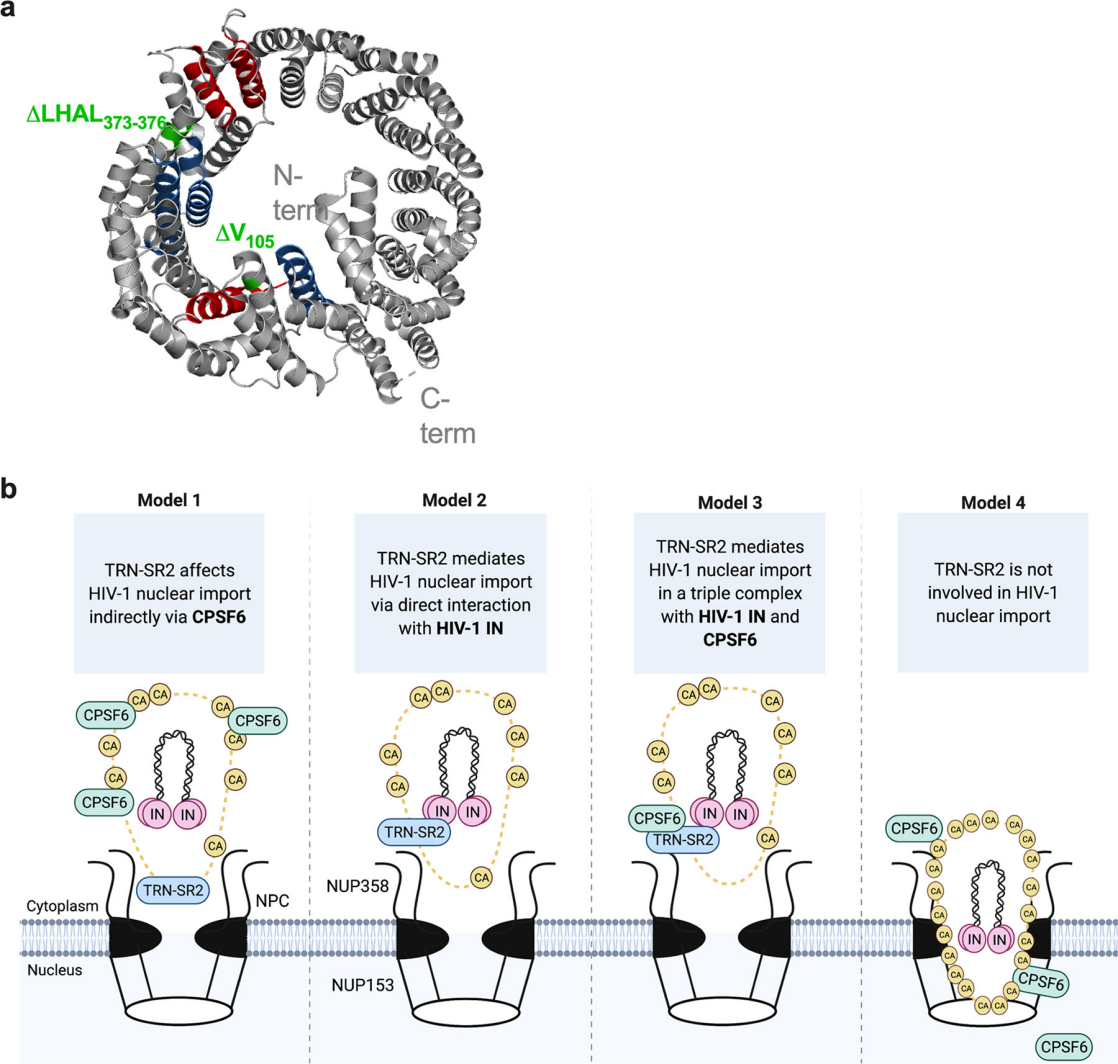
Toward a model of HIV-1 nuclear import. (a) Position of TRN-SR2 peptides interacting with HIV-1 IN in the three-dimensional structure (ribbon) of TRN-SR2 (PDB entry 4C0P) revealed by a previous peptide interaction screen (27). The TRN-SR2 peptides showing strong positive interaction with IN are colored red. The peptides showing interaction with HIV-1 IN are colored blue. Noninteracting and aspecific binders are depicted in gray. The CRISPR/Cas9-induced deletion of Val at position 105 (ΔV_105_) and the deletion of LHAL at position 373 (ΔLHAL_373–376_) are colored in green. (b) Four distinct models of nuclear import of the HIV-1 PIC. The HIV-1 PIC contains the nascent HIV-1 DNA during the reverse transcription process, the HIV-1 IN, and the remaining HIV-1 CA. The PIC interacts with the nuclear pore proteins to achieve active nuclear import. (Model 1) The interaction between the CA and NUP358 engages the PIC to access the nucleus. In this model, the association of TRN-SR2 with the PIC is an artifact of CPSF6 binding to TRN-SR2. (Model 2) TRN-SR2 docks to the FG repeats of the NUPs and interacts with HIV-1 IN. As such, TRN-SR2 mediates nuclear import of the PIC. (Model 3) TRN-SR2 forms a triple complex with both CPSF6 and HIV-1 IN to mediate HIV-1 nuclear import. (Model 4) The above models rely on the assumption that uncoating initiates in the cytoplasm and/or at the NPC and that CA loss from the viral core allows the cellular factors to associate with the PIC. In model 4, cone integrity is retained during nuclear import and the intact cones recruit CPSF6, which facilitates nuclear import of an intact cone. In this case, TRN-SR2 does not play a role in nuclear import of the PIC but may play a role between nuclear import and integration. PIC, preintegration complex; IN, integrase; CA, capsid; NUPs, nuclear proteins; NPC, nuclear pore complex. Figure created with Biorender.com.

Although most research groups agree on a role of TRN-SR2 in HIV-1 replication (4, 8, 11, 13, 16, 20, 35, 39, 40, 43, 44), no consensus exists about its mechanism. According to a first model, TRN-SR2 plays an indirect role via interaction with CPSF6 and uncoating of the viral CA (7, 8) (Fig. 7b, model 1). According to this model, cytoplasmic accumulation of CPSF6 in TRN-SR2 KD cells blocks HIV-1 replication through the interaction with the HIV-1 CA (7, 11, 29). It was indeed shown that CPSF6-induced stabilization of CA in the cytoplasm leads to a delayed CA uncoating and a block in HIV-1 nuclear import (7, 14, 23, 35). However, cytoplasmic relocalization of CPSF6 upon TRN-SR2 depletion has not been demonstrated convincingly (23, 35). Moreover, nuclear import of mRNA processing factors, including CPSF6, is mediated by importins other than TRN-SR2, implying redundancy (45). We did not observe relocalization of CPSF6 in HeLaP4 TRN-SR2 KD or in the HeLaP4 clones. In this study, TRN-SR2 KD did not induce cytoplasmic accumulation of CPSF6 as suggested before (23, 35). The first model of HIV-1 nuclear import is also supported by the N74D CA mutant phenotype. The N74D CA mutation is known to impair the interaction of the HIV-1 CA core with CPSF6, and this mutant replicates independently of TRN-SR2 (11, 13, 16, 38, 39), supporting the model in which TRN-SR2 indirectly mediates nuclear import via CPSF6. Thys et al. confirmed that N74D is insensitive to TRN-SR2 depletion when pseudotyped with VSV-g but retains partial TRN-SR2 dependency with the HIV-1 envelope (13). Moreover, a multiple-round N74D virus strain proved strongly dependent on TRN-SR2 in that study, contradicting the theory that CPSF6 mediates the TRN-SR2 phenotype (8). In this study, we confirmed that the N74D CA HIV-1, in contrast to WT HIV-1, does not require TRN-SR2 during a single-round infection when pseudotyped with VSV-g, as reported by earlier studies (11, 13, 16, 38). In contrast, clone 15.15 and 15.20 were severely impaired for HIV-1 infectivity for both WT and N74D mutant HIV-1 (Fig. 6). Since N74D replicates independently of CPSF6 (11), these data provide further evidence that neither the CA-CPSF6 interaction nor the TRN-SR2-CPSF6 interaction is the primary determinant of TRN-SR2 dependence in our CRISPR/Cas9-induced TRN-SR2 mutants.

To further exclude indirect effects related to the cellular function of TRN-SR2, we investigated whether the TRN-SR2 mutants were defective for nuclear transport of SR domain-containing cargo proteins. These proteins consist of essential pre-mRNA splicing and polyadenylation factors, such as ASF/SF2 (also known as serine/arginine-rich splicing factor 1 [SRSF1]), SR-rich splicing factor 2 (SRSF2), and CPSF6, all proteins that dynamically shuttle between the cytoplasm and the nucleus (46, 47). Using immunocytochemistry, we show that regardless of the induced mutations, TRN-SR2 maintained the ability to import ASF/SF2 and CPSF6 into the nucleus (Fig. 4c and d), which is in agreement with other reports that could not observe cytoplasmic accumulation of CPSF6 or ASF/SF2 after TRN-SR2 depletion (4, 23, 35). Additionally, perturbing levels of ASF/SF2 may dysregulate alternative splicing of the HIV-1 pre-mRNA and therefore interfere with virus progeny production (37). However, no significant difference in multiple-spliced HIV-1 pre-mRNA between control and TRN-SR2 mutant cell lines was observed (Fig. 5). Based on both immunocytochemistry and pre-mRNA splicing data, we conclude that the CRISPR/Cas9-induced TRN-SR2 mutants are not impaired in the import of natural cargoes of TRN-SR2 or pre-mRNA splicing. Hence, the mutations do not negatively interfere with the cellular functions of TRN-SR2. Both the sensitivity of the N74D virus to TRN-SR2 mutations and the lack of effect on cellular cargoes upon TRN-SR2 depletion or mutation refute the theory whereby cytoplasmic CPSF6 explains the TRN-SR2 phenotype in model 1 (Fig. 7b).

Alternatively to the first model, in which TRN-SR2 indirectly facilitates nuclear import, a direct role of TRN-SR2 has been proposed via an interaction with HIV-1 IN (4, 20–22, 27, 28) (Fig. 7b, model 2). Although several studies have shown that TRN-SR2 interacts directly with HIV-1 IN (4, 8, 13, 28), strong evidence that nuclear import of HIV-1 is solely dependent on the physical interaction between these two proteins is absent. Earlier studies pinpointed the interface between TRN-SR2 and HIV-1 IN to the N terminus of TRN-SR2, predominantly via the HEAT repeats 4, 10, and 11 (27). According to this model, HIV-1 IN binding does not interfere with binding of SR-rich cargoes to the C terminus of TRN-SR2. Using size exclusion chromatography, Tsirkone et al. evidenced a triple complex whereby TRN-SR2 engages nuclear import of HIV-1 IN in parallel with SR-rich cargoes (27). Nuclear Ran-GTP would promote the release of both the endogenous cargo and HIV-1 IN (48) upon arrival in the nucleus. Evidence for a direct interaction between TRN-SR2 and HIV-1 IN is difficult to obtain. Unfortunately, mutations in HIV-1 IN are notoriously pleiotropic and interfere with other crucial steps in viral replication, such as reverse transcription, integration, or assembly, confounding interpretation of results. Cribier et al. could not corroborate a nuclear import defect for some HIV-1 IN mutants deficient for TRN-SR2 binding (43). Still, this study investigated only the effect on 2-LTR circles as indirect measurement for nuclear import, and the tested mutants were also deficient for interaction with LEDGF/p75 (43). Interference with both nuclear import and integration may induce opposite effects on the level of 2-LTR circles, obscuring an effect on nuclear import. De Houwer et al. introduced mutations in the C terminus of HIV-1 IN that abrogated the binding with TRN-SR2 (21). By employing a PIC nuclear import assay with IN-eGFP-labeled virus, the authors provided clear evidence that mutations in HIV-1 (IN^R263A/K264A^) at the interface with TRN-SR2 impaired HIV-1 replication at the level of nuclear import (21).

Here, we rely on the ΔV_105_ TRN-SR2 mutant that is partially defective for HIV-1 IN interaction to corroborate the direct link between the TRN-SR2/IN interaction and HIV-1 nuclear import (Fig. 7b, model 2). To prove a direct link, mutations in TRN-SR2 should decrease the interaction with HIV-1 IN without affecting its normal cellular function as a cellular import factor. Although in this study we initially opted to knockout TRN-SR2, our strategy serendipitously yielded at least one TRN-SR2 mutant defective for interaction with HIV-1 IN but without effect on the cellular function of TRN-SR2. Our CRISPR/Cas9-induced clones were selected based on reduced TRN-SR2 expression and on resistance to HIV-1 infection, which eventually resulted in the selection of a TRN-SR2/IN interface mutant in clone 15.15. TRN-SR2 expressed in clone 15.15 (ΔV_105_ TRN-SR2) displays a 2-fold reduction in HIV-1 IN binding, consistent with a 2-fold reduction in HIV-1 replication and nuclear import (Fig. 2c and d, Fig. 3d, and Fig. 4a). The additional 4-amino-acid deletion at position 373 in clone 15.20 (ΔV_105_ ΔLHAL_373–376_ TRN-SR2) affected HIV-1 replication and nuclear import more severely compared to the single deletion of Val at position 105, but it was not possible to purify the respective recombinant protein expressed in clone 15.20, which may be due to poor stability of Escherichia coli-produced proteins (e.g., protein misfolding or the absence of posttranslational modifications). Still, as outlined in Figure 7a, the additional deletions at position 373 are present in the region of the proposed interface with HIV-1 IN (20), and therefore, we might argue that the additional deletions affected the interaction with IN even more profoundly, resulting in an additional interface mutation. Overall, these data support a model in which nuclear import of HIV-1 is dependent on the physical interaction between TRN-SR2 and HIV-1 IN (Fig. 7b, model 2).

The direct role of TRN-SR2/IN interaction in nuclear import does not exclude that CPSF6 indirectly affects nuclear import of the PIC. According to the triple complex model proposed by Tsirkone et al. (27), TRN-SR2 can mediate simultaneous import of HIV-1 IN and natural SR cargoes. Therefore, mutations in TRN-SR2 that reduce the interaction with HIV-1 IN do not necessarily affect binding of CPSF6 and hence its nuclear import. Indeed, our CRISPR/Cas9 approach induced mutations in the N-terminal Ran-GTP binding region (1 to 281) and the central region of TRN-SR2 (281 to 531), both regions of the proposed interface with HIV-1 IN (20), while the C-terminal cargo binding region was not affected. According to this model (Fig. 7b, model 3), nuclear import is facilitated by TRN-SR2-mediated import of HIV-1 IN as part of the PIC. While HIV-1 IN interacts with the N-terminal half of TRN-SR2, CPSF6 (or ASF/SF2) can be imported through an interaction with the cargo domain. In agreement with this model, the CRISPR/Cas9-induced TRN-SR2 clones used in this study are not impaired in nuclear import of CPSF6 and ASF/SF2 (Fig. 4c and d), while HIV-1 nuclear import is impaired.

Few authors have proposed a mechanism by which TRN-SR2 mediates HIV-1 nuclear import through direct interaction with CA (8, 35, 40). The fact that the mechanism and kinetics of CA uncoating are still heavily debated adds to the unknowns of HIV-1 nuclear import. Until recently, HIV-1 uncoating, which is defined as the loss of viral CA from the PIC, was believed to initiate in the cytoplasm and/or at the nuclear pore complex (49–52), making the nascent PIC accessible to host factor interactions during nuclear import. Recent studies provide an alternative hypothesis in which the viral core remains intact during nuclear import and as a consequence uncoating initiates only within the nucleus (53, 54). According to this model (Fig. 7b, model 4), TRN-SR2 cannot interact with the nascent PIC at the nuclear pore complex and thus would play no role in nuclear import of the PIC. However, whether an intact core can pass the nuclear pore complex without shearing/uncoating is still matter of debate. Model 4 could still be consistent with a postnuclear entry role of TRN-SR2 as proposed before (40).

Although CRISPR/Cas9 has been developed to generate knockout alleles, the nonhomologous end joining (NHEJ) DNA repair mechanism following Cas9 cleavage at the target site often induces frameshift mutations resulting in nonfunctional or mutant proteins. In fact, CRISPR/Cas9-induced phenotypic selection of mutant cell lines has been used to screen for host-pathogen interactions in infectious diseases (55, 56) or to identify genes important for cell proliferation in cancer (57). The use of gRNA libraries (58) that target the entire human genome allows high-throughput targeting and identification of genes as potential host factors in viral infections. Important host factors of hepatitis C virus and Zika virus have been discovered using CRISPR/Cas9-induced screens (55). This approach is of particular interest when studying host factors that have an essential role in the cell biology. Mutations at host-pathogen interfaces will confer resistance to the viral pathogen, while mutations that affect the structural integrity and biological function of essential host proteins will not survive the selection. In this study, we selected at least one TRN-SR2 mutation at the interface with HIV-1 IN blocking HIV-1 infection without affecting the cellular function of TRN-SR2. HIV-1 and other viruses in general heavily rely on host proteins to complete their replication cycle (59). This study provides a novel CRISPR/Cas9-based editing method to target and detect hot spot interactions of host-pathogen protein-protein interfaces.

Overall, this study addressed the unanswered question on the role of TRN-SR2 in HIV-1 nuclear import by employing CRISPR/Cas9 selective gene targeting to generate interface indel mutants of TRN-SR2. The presented data provide evidence that TRN-SR2 is directly involved in nuclear import via its interaction with HIV-1 IN irrespective of the TRN-SR2-mediated nuclear import of CPSF6. Furthermore, we demonstrate that an HIV-1 IN interface mutant of TRN-SR2 allows to specifically reduce HIV-1 nuclear import without affecting the cellular functions of TRN-SR2. Our finding opens perspectives to target nuclear import, a bottleneck in the HIV-1 life cycle without clinically available inhibitors.

## MATERIALS AND METHODS

### Cell culture.

HeLaP4 cells were cultured in Dulbecco’s modified Eagle medium (DMEM) supplemented with 5% fetal bovine serum (FBS), 50 μg/ml gentamicin, and 500 μg/ml Geneticin. The cells were cultured in a humidified atmosphere containing 5% CO_2_ at 37°C. For selection and maintenance of HeLaP4 cell clones after lentiviral transduction, appropriate antibiotics were used at the following concentrations: puromycin (1 μg/ml) and hygromycin B (2 μg/ml, Invitrogen). Unless otherwise specified, all cell culture media and reagents were obtained from Gibco Thermo Fisher Scientific.

### Recombinant DNA and CRISPR/Cas9 vectors.

Guide RNAs (gRNAs) were designed by the CRISPR design and analysis tool from the Massachusetts Institute of Technology (http://crispr.mit.edu). gRNAs were generated by annealing of the forward and reverse primers. Primer sequences are shown in Table S1. The gRNA-coding plasmids were cloned by BbsI-mediated insertion of the annealed products into the pX321-eGFP plasmid backbone (60). The plasmid encoding the Cas9 transfer plasmid was derived from pLenti-CRISPR-v2 (Addgene). Adaptors were inserted in the plasmid, together with a puromycin resistance cassette, as described previously (60). For lentiviral vector production, cotransfection of the Cas9 transfer plasmid (pLenti-CRISPR-v2 plus adaptor), the p8.91 packaging plasmid, and pVSV-g was performed as described previously (60). The resulting lentiviral vector (LV) is referred to here as LV_Cas9-I-PuroR. For TRN-SR2 back-complementation, a lentiviral vector was produced by transfecting HEK-293T cells with the transfer construct (pCHMWS-TRN-SR2-BC-IRES-Hygro), together with the p8.91 packaging (61) and VSV-g envelope expressing plasmids. Branched polyethylenimine (bPEI, 10 μM, Sigma-Aldrich) was used for plasmid transfections. Medium was replaced 6 h posttransfection, and supernatant was collected after 48 h and 72 h by filtration through a 0.45-μm pore-size filter and concentrated by ultrafiltration (Amicon Ultra-15 centrifugal filter unit, 50 kDa, Merck).

### Generation of stable cell lines.

Generation of mutant TRN-SR2 clones is outlined in Figure 1b. First, stable HeLaP4-Cas9-expressing cells were generated by lentiviral vector transduction and subsequent puromycin selection. Stable polyclonal populations were expanded and used in downstream experiments. Freshly seeded HeLaP4-Cas9 cells were subsequently electroporated with pX321-eGFP plasmid containing *TNPO3*-specific gRNA2 and 8, and monoclonal antibodies were seeded 3 days later as described earlier (60). Following electroporation, cells were sorted for GFP using fluorescence activated cell sorting (Bio-Rad S3) and were allowed to expand for 1 week before seeding for monoclonal expansion into a 96-well plate. The individual HeLaP4 clones were screened by Western blotting for TRN-SR2 protein level compared to that of HeLaP4 TRN-SR2 KD cells (Fig. S1a). Two clones, clone 2 and clone 15, were selected and evaluated at the genomic DNA level indicating monoallelic deletion at the gRNA2 target position. A second round of gRNA8-pX321-eGFP electroporation followed by monoclonal selection was initiated specifically for clone 15, which showed the most promising growth characteristics. Five clones were selected for further work based on their TRN-SR2 protein levels. To back-complement wild-type TRN-SR2 (TRN-SR2^WT^), the selected clones were transduced with the pCHMWS-TRN-SR2-BC-IRES-Hygro vector and polyclonal populations were selected in hygromycin-containing medium. The TRN-SR2 stable KD and scrambled control vector (SCR) cells were described previously (13).

### Sequencing of genomic DNA and mRNA.

To map the edits induced by CRISPR/Cas9 action, genomic DNA and mRNA (cDNA) were sequenced following amplification of the regions flanking the gRNA2 and gRNA8 targets. For gDNA sequencing, DNA was first isolated using Sigma mammalian genomic DNA miniprep kit (Sigma-Aldrich). The gDNA region flanking exon 2 was sequenced using MiSeq primers (Fw: tcgtcggcagcgtcagatgtgtataagagacagcaggttcatgcatgggagatctc; Rev: gtctcgtgggctcggagatgtgtataagagacagcctcacatggcctagcaatacg), performed as described previously (60). The *TNPO3* genomic region corresponding to exon 8 was first PCR-amplified and the PCR fragments were cloned in the pJET plasmid (Thermo Scientific) and sequenced with the following primers: Fw, cgactcactatagggagagcggc and Rev, aagaacatcgattttccatggcag. For cDNA sequencing, RNA was extracted using the Aurum total RNA minikit (Bio-Rad) and cDNA was generated using the high-capacity cDNA reverse transcription kit (Thermo Scientific). Next, specific amplicons were generated by PCR: exon 2 (Fw: gcaggatgtggagtcatgc; Rev: ttccaggaaggcatctgtagg) and exon 8 (Fw: gcttatctgtgcaggccatcct; Rev: ctgataccctcatgcgaaactcc).

### Cell growth analysis.

To compare the growth and cell division characteristics of all HeLaP4 cells used in this study, we counted the cells over six culture passages in T75 cell culture flasks. The cells were washed with phosphate-buffered saline (PBS), trypsinized, and then resuspended in DMEM supplemented with 5% FBS and appropriate antibiotics. Cells in suspension were then counted with the Z1 Coulter particle counter (Beckman Coulter).

### Nucleic acid extraction and reverse transcriptase quantitative PCR (RT-qPCR).

To determine *TNPO3* mRNA levels, RNA was extracted from 2 × 10^6^ cells using the Aurum total RNA minikit (Bio-Rad). A total of 5 μg of total RNA was reverse transcribed to cDNA, as described previously (13). We used two sets of primers and probes: 5′-terminal set (Fw: gccattagccatgcaacttt; Rev: aagcagcagctccagagttc; probe: gctctgggagatcatgcagg) and 3′-terminal set (Fw: ctaccagatgtggctgaagt; Rev: acaaaaagtcggtctgtcaa; TaqMan probe: gctctgggagatcatgcagg). TRN-SR2 cDNA amounts were normalized to β-actin cDNA levels (Fw: cactgagcgaggctacagctt; Rev: ttgatgtcgcgcacgattt; TaqMan probe: accaccacggccgagcgg). The 20 μl master mix per sample consisted of 12.5 μl IQ super mix (Bio-Rad), 1 μl of each 5 μM primer/5 μM probe (for TRN-SR2), 0.5 μl of each 5 μM primer/5 μM probe (for β-actin), and water. Per reaction, 5 μl of cDNA was added. The samples were run in triplicate under the following cycling conditions: 95°C for 3 min, 50 cycles of 95°C for 10 s, and 55°C for 30 s. All products were quantified using Roche light cycler 480 software (Roche).

Multiple-spliced viral mRNA was quantified as described previously (4). In short, 5 × 10^5^ cells were transfected with a replication-competent NL4.3-based plasmid. After 24 h of plasmid transfection, the mRNA was isolated (total RNA minikit) and reverse transcribed (high-capacity cDNA archive kit). Quantitative PCR was performed using Sybr green PCR master mix (Applied Biosystems) with forward and reverse multiple-spliced primers (Fw: ggcttgctgaagcgcgcacggcaagagg; Rev: ttggaggtgggttgctttgatagag). The samples were normalized to β-actin cDNA levels as described above.

### Quantification of late RT products, 2-LTR circles, and integrated viral DNA.

A total of 5 × 10^5^ cells were seeded and treated with dimethyl sulfoxide (DMSO) or the inhibitors azidothymidine (AZT) or raltegravir (Ral) prior to transduction. Four hours posttransduction with replication-deficient HIV-1 NL4.3, the cells were washed three times with PBS and fresh medium, supplemented with DMSO or compound, was replenished on the cells. The cells were harvested at 8, 10, 12, 24, or 48 h posttransduction and DNA was extracted using the Sigma mammalian genomic DNA miniprep kit (Sigma-Aldrich). The isolated DNA was subjected to qPCR to quantify late RT (Fw: tgtgtgcccgtctgttgtgt; Rev: gagtcctgcgtcgagagagc; TaqMan probe: gcagtggcgcccgaacaggga) and 2-LTR viral DNA products (Fw: gtgcccgtctgttgtgtgact; Rev: cttgtcttctttgggagtgaattagc; TaqMan probe: tccacactgactaaaagggtctgagggatctct). The late RT and 2-LTR circle copy numbers were normalized for β-actin content (Fw: tcacccacactgtgcccatctacga; Rev: cagcggaaccgctcattgccaatgg; TaqMan probe: atgccctcccccatgccatcctgcgt). The 20 μl master mix per sample consisted of 12.5 μl IQ super mix (Bio-Rad), 1.5 μl of each 5 μM primer, 1 μl of 5 μM probe (for late RT and 2-LTR), and 1 μl of each 5 μM primer/5 μM probe (β-actin) and water. A standard curve was generated and no-template controls were included. The samples were run in duplicate under the following cycling conditions: 95°C for 5 min and 50 cycles of amplification (10 s at 95°C, 30 s at 55°C). All products were quantified using Roche light cycler 480 software (Roche). To quantify integrated HIV-1 DNA, 2 × 10^4^ cells were seeded per well in 96-well plates and transduced the following day with single-round HIV-1 expressing firefly luciferase. DNA was extracted 10 days after transduction using Sigma mammalian genomic DNA miniprep kit (Sigma-Aldrich). The isolated DNA was subjected to qPCR to quantify integrated HIV-1 DNA (Fw: gaagagatacgccctggttcc; Rev: tgtgatttgtattcagcccatatcg; TaqMan probe: ttcatagcttctgccaaccgaacggaca). The copy number was normalized for CCR5 content (Fw: gctgtgtttgcgtctctcccagga; Rev: ctcacagccctgtgcctcttcttc).

### Western blotting.

Cell pellets were obtained from 1 × 10^6^ cells and dissolved in 300 μl 1% (wt/vol) SDS. The lysed cells were boiled at 95°C for 5 min and the lysates were subsequently homogenized by repeated pipetting through a 0.3-mm insulin needle. Protein concentration was determined by the bicinchoninic acid (BCA) assay (Thermo Scientific Pierce). A total of 30 μg of protein extract was loaded and separated on a 7.5% SDS-PAGE gel. The proteins were transferred onto a polyvinylidene difluoride (PVDF) membrane (Bio-Rad) using a Turbo blotting protocol of the manufacturer. The membrane was incubated at 4°C overnight with primary antibodies: rabbit α-TRN-SR2 (ab 109386, Abcam) and mouse α-beta-tubulin (ab 14545, Abcam). After washing with PBS-T (PBS with 0.1% Triton-X-100), the membrane was incubated with the secondary antibody for 1 h at room temperature. The secondary horseradish peroxidase (HRP)-conjugated antibodies used were goat anti-mouse and goat anti-rabbit, both diluted 1:3,000 in blocking buffer (5% [m/vol] milk). The secondary antibody was washed away and stained proteins were detected by chemiluminescence (Clarity western ECL, Bio-Rad). The Western blot signal was quantified using Fiji software. Percentage of protein expression was calculated as a ratio of each protein band relative to the lane’s loading control.

### Single-round transduction with HIV-1-fLuc.

The viral molecular clone pNL4-3.Luc.RE- was obtained through the NIH AIDS Research and Reagent Reference program and was used to produce HIV-1 replication-deficient luciferase reporter virus. The construct pNL4-3.Luc.RE- N74D CA encoding an N74D CA mutant reporter virus was a kind gift of Vineet KewalRamani (National Cancer Institute, Maryland). Replication-deficient (here referred to as “single-round”) virus was prepared by cotransfection of HEK-293T cells with pNL4-3.Luc.RE- or pNL4-3.Luc.RE- N74D CA and pVSV-g. One day prior to transduction, 2 × 10^4^ cells were seeded per well in 96-well plates. The following day, the cells were transduced in 3- or 5-fold serial dilutions with single-round virus. At 72 h posttransduction, cells were pelleted and lysed in buffer (50 mM Tris, 200 mM NaCl, 0.2% [vol/vol] NP-40, and 5% [vol/vol] glycerol) and subsequently analyzed for firefly luciferase activity (ONE-Glo Promega GMBH, Mannheim, Germany). Chemiluminescence was measured with a Glomax luminometer (Promega). Readouts were normalized for protein content as determined by BCA assay (Thermo Scientific Pierce).

### Breakthrough assay.

To evaluate the kinetics of viral breakthrough, cells were infected with 2 × 10^4^ pg p24 replication-competent HIV-1 NL4.3. Virus production was quantified on successive days by measuring HIV-1 capsid (p24) protein by ELISA in the supernatant (INNOTEST p24-ELISA, Innogenetics). p24 enzyme-linked immunosorbent assay (ELISA) was performed according to the manufacturer’s instructions.

### Confocal microscopy imaging of intracellular ASF/SF2, CPSF6, and TRN-SR2.

For immunocytochemistry analysis, 3 × 10^5^ cells were seeded in 8-well chamber slides (Nunc Lab-Tek Chambered Coverglasses, 155411, Thermo Scientific). The next day, the cells were fixed with 4% (vol/vol) paraformaldehyde, permeabilized using 0.1% Triton-X-PBS. Monoclonal antibodies for ASF/SF2 (sc-28724, Santa Cruz), CPSF6 (HPA039973, Atlas), or TRN-SR2 (EPR5264, Abcam) were used at a concentration of 1:50, 1:250, and 1:1,000, respectively. Secondary antibody was conjugated to Alexa 488 (ASF/SF2 and TRN-SR2) or Alexa 555 (CPSF6), and DAPI (4′,6-diamidino-2-phenylindole; 0.001 μg/μl) was used for nuclear staining (pseudocolored blue in the images). Images were acquired using the UPLSAPO 60× W NA 1.25 objective and the DM405/488/559/635 polychromic excitation mirror (Olympus).

### PIC nuclear import assay.

The generation of HIV-1 particles containing fluorescently labeled IN (IN-eGFP) by Vpr-mediated *trans*-incorporation and the HIV-1 nuclear import assay were performed as described previously (21). One day prior to transduction, 25 × 10^3^ cells were seeded in 8-well chamber slides (Nunc Lab-Tek chambered cover glasses, 155411, Thermo Scientific), previously coated with poly-d-lysine (0.1 mg/ml, Sigma). Cells were fixed 6 h after transduction for 15 min with 4% (vol/vol) paraformaldehyde and permeabilized for 5 min with 0.1% (vol/vol) Triton-X-100. Nuclei were immunostained with lamin A/C antibody (mouse monoclonal, 1/400 dilution, sc-7292, Santa Cruz Biotechnology) and secondary anti-mouse IgG(H+L) Alexa 405 conjugate (goat polyclonal, 1/500 dilution, A-10011, Life Technologies) both diluted in blocking buffer (1% [wt/vol] bovine serum albumin [BSA] and 0.1% [vol/vol] Tween 20 in PBS). The cells were imaged using a laser scanning microscope (Fluoview FV1000, Olympus, Tokyo, Japan). Images were acquired using the UPLSAPO 60× W NA 1.25 objective and the DM405/488/559/635 polychromic excitation mirror (Olympus). The IN-eGFP complexes are pseudocolored green in the images and lamin is pseudocolored blue in the images. Fixed cells and virus particles were imaged in 3-dimensionsal (3D) stacks, with a 0.3-μm step size and 4 μs/pixel sampling speed. An in-house MATLAB routine (MatWorks Inc.) was used to determine the localization and number of IN-eGFP complexes, as described previously (33). In short, IN-eGFP complexes and the nuclear lamin were identified automatically using an intensity threshold. A fluorescent spot was assigned as a PIC if at least two connecting pixels were above the threshold and if the fluorescent signal was present in at least two consecutive frames (z planes). Based on the nuclear lamin staining, IN-eGFP complexes were divided into cytoplasmic or nuclear compartments and the percentage of nuclear IN-eGFP complexes was calculated.

### Recombinant protein purification.

Full-length ASF/SF2 was cloned into pMAL-c2E (Addgene) using EcoRI and SAII sites. Cultures of E. coli strain BL21 transformed with pMal-ASF/SF2 were grown to an optical density at 600 nm (OD_600_) of 0.6 in LB supplemented with 2 g/liter glucose and induced overnight at 16°C with 0.5 mM isopropyl-d-thiogalactoside. The cultures were harvested by centrifugation for 10 min at 4,000 rpm at 4°C, and pellets were washed with buffer (100 mM Tris-HCl [pH 7.5], 1 M NaCl, and 1 mM EDTA), centrifuged again, and stored at −80°C until purification. The frozen cultures were thawed, resuspended in lysis buffer (50 mM Tris-HCl [pH 7.5], 350 mM NaCl, 2 units DNase/10 ml, and 1 tablet Complete protease inhibitor/50 ml [Roche]) and lysed by sonication, and the lysate was centrifuged at 15,000 rpm at 4°C for 30 min. The soluble lysate containing maltose-binding protein (MBP)-fused protein was loaded onto an amylose resin column (New England BioLabs) equilibrated with binding buffer (50 mM Tris-HCl [pH 7.5], 350 mM NaCl). The protein was eluted with the elution buffer [50 mM Tris-HCl (pH 7.5), 350 mM NaCl, 10 mM D(+) maltose] followed by overnight dialysis in buffer (50 mM Tris-HCl [pH 7.5], 350 mM NaCl, 10% glycerol) and stored at −80°C. N-terminally His_6_-tagged HIV-1 IN (62), glutathione transferase (GST)-tagged ASF/SF2 (48), HIS_6_-tagged TRN-SR2 (27), and GST-tagged TRN-SR2 (48) were purified as described previously. Site-directed mutagenesis (New England BioLabs) was used to introduce the ΔV at position 105 (Fw: acgcagctggctttagca; Rev: aataacaggtgacaagtctttcaagttc) and the ΔLHAL deletion at position 373 (Fw: ggctcgacactgccagct; Rev: agcctctgaatgtaagctttgaagatg) in GST-tagged and His_6_-tagged TRN-SR2 constructs.

### AlphaScreen protein-protein interaction assay.

The AlphaScreen binding assay was performed as described previously (20). In brief, proteins were all diluted to 5× working solutions in the assay buffer (25 mM Tris [pH 7.4], 150 mM NaCl, 1 mM MgCl_2_, 0.1% [vol/vol] Tween 20, and 0.1% [wt/vol] BSA). To determine the interaction between TRN-SR2 and IN, 10 μl of IN (GST-IN or His_6_-IN at a final assay concentration of 80 nM and 200 nM, respectively) was first pipetted into the wells, followed by 5 μl of a TRN-SR2 dilution series (His_6_-TRN or GST-TRN, in a 3-fold dilution series starting at 1,000 nM). The plate was sealed and left to incubate for 1 h at 4°C. Next, 10 μl of a mixture of Ni2^+^ chelate acceptor and glutathione donor AlphaScreen beads (PerkinElmer Life Sciences) was added, at a final concentration of 0.02 mg/ml for each of the beads. Plates were then incubated for 1 h at 30°C and analyzed using an EnVision multilabel reader (PerkinElmer Life Sciences) according to the manufacturer’s instructions. To determine the interaction between TRN-SR2 and ASF/SF2, 10 μl of ASF/SF2 (GST-ASF or MBP-ASF, at a final assay concentration of 30 nM and 10 nM, respectively) was first pipetted into the wells, followed by 5 μl of a TRN-SR2 dilution series (His_6_-TRN or GST-TRN, in a 3-fold dilution series starting from 1,000 nM). A mixture of MBP acceptor and glutathione donor AlphaScreen beads (PerkinElmer Life Sciences) was used. Each titration was performed in duplicate, and assays were repeated at least twice in independent experiments.

### RNAscope technology.

Multiple-spliced viral mRNA was determined with RNAscope technology (Advanced Cell Diagnostics) and performed according to the manufacturer’s instructions (63). Briefly, 1.5 × 10^6^ cells plated on a collagen-coated coverslip (Neuvitro) were transfected with a replication-competent NL4.3-based plasmid. After 24 h of plasmid transfection, the cells were fixed with 4% (vol/vol) paraformaldehyde and dehydrated using increasing concentrations of ethanol (0, 50, 70, 100% [vol/vol]). After dehydration, samples were stored in 100% ethanol at −20°C. To rehydrate the cells, decreasing concentrations of ethanol were used (70, 50, 0% [vol/vol]). Following rehydration, the cells were permeabilized using 0.1% Tween 20 in PBS. Unspliced viral RNA was detected with the RNA probe targeting the gag/pol region of the HIV-1 genome (317691-C2, Advanced Cell Diagnostics). Total viral RNA (unspliced and multiple-spliced viral RNA) was detected with the nongag/pol probe (317711-C2, Advanced Cell Diagnostics). For fluorescent detection, the probe was conjugated to the Atto 550 nm label (pseudocolored green in the images) and DAPI (0.001 μg/μl) was used for nuclear staining (pseudocolored blue in the images). The cells were imaged using a laser scanning microscope (Fluoview FV1000, Olympus, Tokyo, Japan) and the images were acquired using the UPLSAPO 60× W NA 1.25 objective and the DM405/488/559/635 polychromic excitation mirror (Olympus). 3D stacks were acquired with a 0.3-μm step size and 4 μs/pixel sampling speed. An in-house MATLAB routine (MatWorks Inc.) was used for vRNA particle detection. Briefly, particles were detected per measured z-stack or frame using an intensity threshold and particle diameter in pixels.

### Statistical analysis.

We used GraphPad Prism 8.4.0 Software Inc. (San Diego, CA, USA) for the statistical analysis. Differences were considered statistically significant for the following *P* values: *, *P* < 0.05; **, *P* < 0.01; ***, *P* < 0.001; ****, *P* < 0.0001.
