# Supplementary material for: CRISPR/Cas9-Induced Mutagenesis Corroborates the Role of Transportin-SR2 in HIV-1 Nuclear Import
Source: Microbiol Spectr. 2021 Oct 6;9(2):e01336-21. doi: 10.1128/Spectrum.01336-21 (PMC8510174; doi:10.1128/Spectrum.01336-21)
Supplement: Supplemental file 1 — Supplemental material. Download SPECTRUM01336-21_Supp_1_seq9.pdf, PDF file, 0.4 MB [file spectrum01336-21_supp_1_seq9.pdf]

Supplemental figures and legends

Table S1. Primer design to generate guide RNAs

gRNA		Primer	
		sense	antisense
1	5′-CCAGTTGTTACAGATCCGGC-3	5′-GCTCTCATGCCTTCCTAGAATGAGTCAG-3′	5′-GTTAACATTAC CTCACATGGCCTAGCAATACG-3′
2	5′-GTCACCTGTTATTGTAACGC-3′	5′-GCTCTCATGCCTTCCTAGAATGAGTCAG-3′	5′-GTTAACATTA CCTCACATGGCCTAGCAATACG-3′
3	5′-TGTTTGCACACATCCCTTCC-3′	5′-ACTGTTGGCTGAATTACTGGGTAATTTAGC-3′	5′-CTCTGTGTAGGT TAATGGCAATTCCAAATGC-3′
8	5′-TCAGAGGCTGCTTCACGCCT-3′	5′-CCAAAGTGCTGGACTACAGG-3′	5′-ACGTGGGAAGACAATTTGGG-3′

Guide RNAs (gRNAs) targeting exon 1, 2, 3 and 8 were generated by annealing of the sense and antisense primers, followed by insertion of the annealing products into the pX321-1 plasmid backbone.

Figure S1. Characterization of clones selected for screening

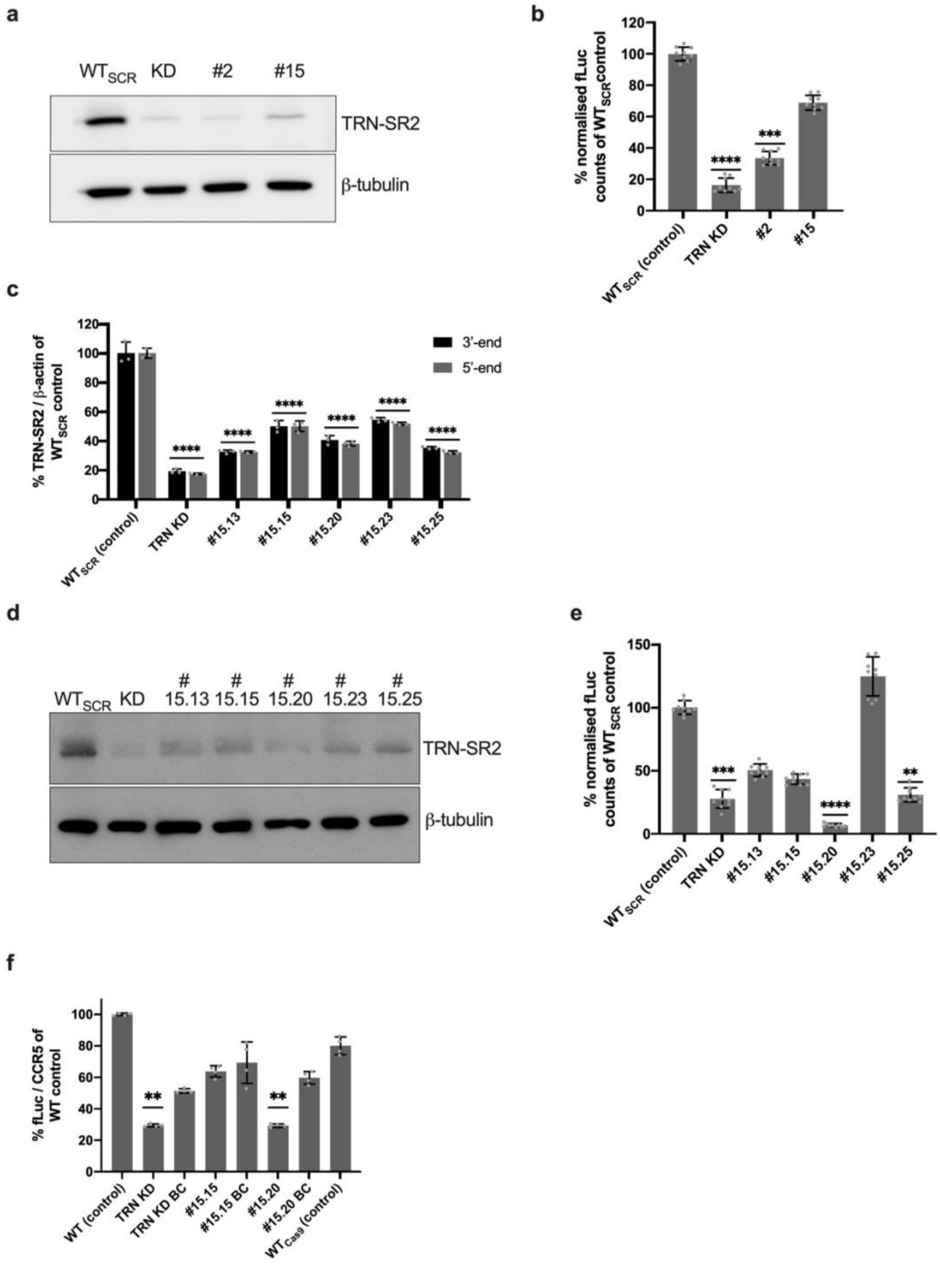

(a, b) TRN-SR2 protein levels were determined by Western blot in control (HeLaP4 WT_{SCR}), HeLaP4 TRN-SR2 KD and **(a)** original clones HeLaP4#2 and #15 and **(b)** clones derived from the population of the maternal HeLaP4#15, using TRN-SR2 specific antibody and a beta-tubulin antibody as the loading control. Western blot signals were quantified with densitometry (Fiji). **(c)** The levels of TRN-SR2 mRNA determined by RT-qPCR,

using primers specific for either the 5'end or the 3'end of TRN-SR2 (exact sequences described in the materials and methods). Mean and standard deviation of two independent experiments, each performed in triplicate, are presented. A repeated measures two-factor ANOVA test was done to test for statistical significance compared to WT_{SCR} control: **** $p < 0.0001$. **(d, e)** Control (HeLaP4 WT_{SCR}), HeLaP4 TRN-SR2 KD and **(d)** original clones HeLaP4#2 and #15 and **(e)** clones derived from the population of the maternal HeLaP4#15, were infected with single round virus expressing firefly luciferase. Luciferase activity was measured 72 h post-infection and normalized to the total protein content. Data from three different virus dilutions (1/1, 1/3 and 1/9) are represented as relative infectivity compared in WT_{SCR} control. Mean and standard deviation of one of two representative experiments, performed in triplicate, is presented. A Kruskal-Wallis test was done to test for statistical significance compared to HeLaP4 WT_{SCR} control: ** $p < 0.01$; *** $p < 0.001$; **** $p < 0.0001$. **(f)** Control (HeLaP4 WT and WT_{Cas9}), HeLaP4 TRN-SR2 KD, HeLaP4 clones and back-complemented cells were infected with single round HIV-1 expressing firefly luciferase and harvested at day 10 post infection to determine total integrated HIV-1 DNA. Data from two different virus dilutions (1/900 and 1/2700) are represented relatively to HeLaP4 WT control. Mean and standard deviation of one of two representative experiments, performed in duplicate, is presented. A Kruskal-Wallis test was done to test for statistical significance compared to HeLaP4 WT control: ** $p < 0.01$.

Figure S2. Cell growth

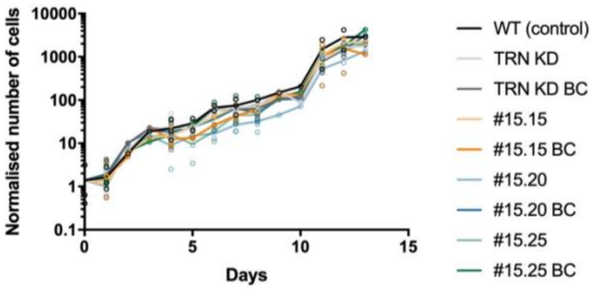

Growth and cell division characteristics of all HeLaP4-derived clones. Cells were seeded and counted on successive days over six passages using the Z1 Coulter Particle Counter (Beckman Coulter).

Figure S3. TRN-SR2 expression levels determined by immunocytochemistry

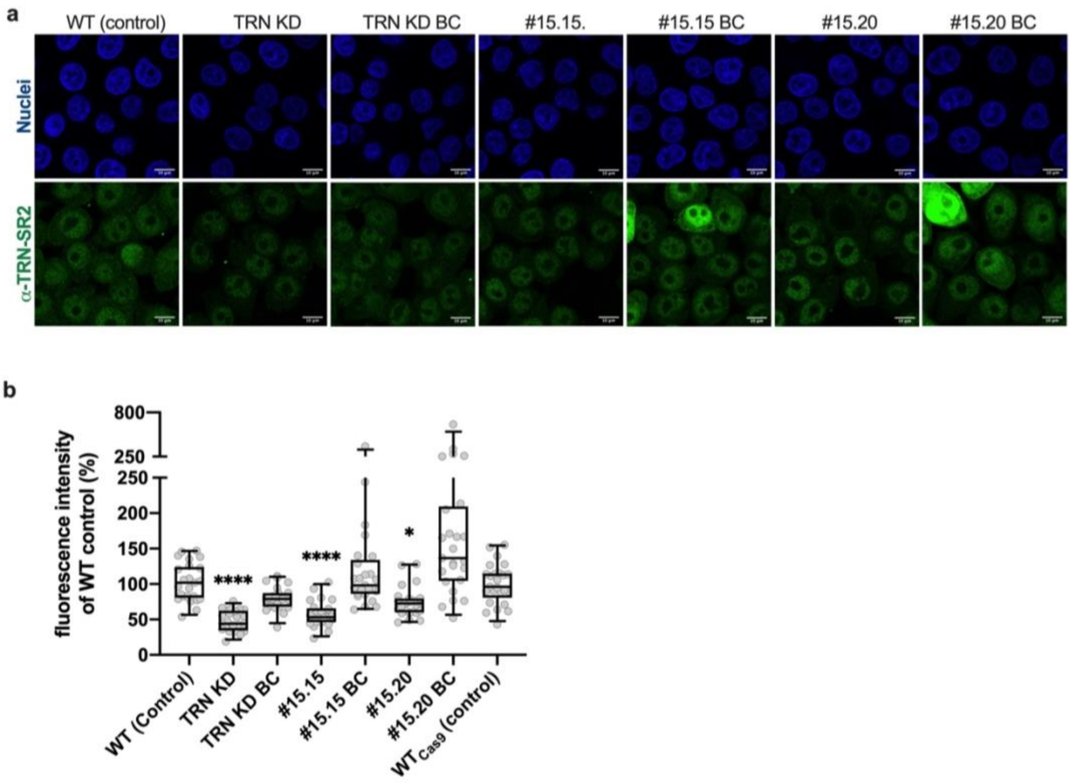

(a) HeLaP4 WT, HeLaP4 TRN-SR2 KD, HeLaP4 clones and back-complemented cell lines were fixed and immunostained with monoclonal antibodies against TRN-SR2. Secondary antibody was conjugated to Alexa 488 (pseudocolored green in the images) while the nucleus was stained with DAPI (pseudocolored blue in the images). Scale bars represent 10 µM. One representative experiment, including the parallel analysis of all cell lines, of two experiments is shown. **(b)** The fluorescence intensity per cell (n = 25 cells per condition) was analyzed with Fiji and plotted relatively to HeLaP4 WT control. 5-95% box-and-wisker plots overlapping individual points are shown from one representative, of two independent experiments. A Kruskal-Wallis test was done to test for statistical significance compared to HeLaP4 WT control: * $p < 0.05$; **** $p < 0.0001$.

Figure S4. 2-LTR circles in back-complemented TRN-SR2 mutant clones

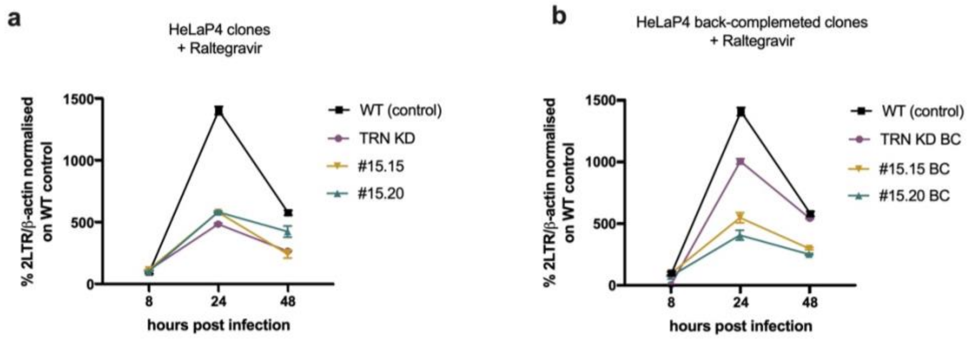

(a, b) Analysis of nuclear import (2-LTR circles) by qPCR. Cells were infected with replication-deficient HIV-1 NL4.3 and harvested at distinct time points after infection. In all conditions, integration was inhibited by raltegravir. The number of 2-LTR circles are shown for **(a)** control (HeLaP4 WT), HeLaP4 TRN-SR2 KD and HeLaP4 clones (#15.15 and #15.20) and **(b)** control (HeLaP4 WT), TRN-SR2 KD BC and back-complemented clones (#15.15 BC and #15.20 BC). Data are represented as means ± SD from one of two representative experiments, performed in duplicate.

Figure S5. CRISPR/Cas9-induced mutagenesis generates a TRN-SR2/IN interface mutant

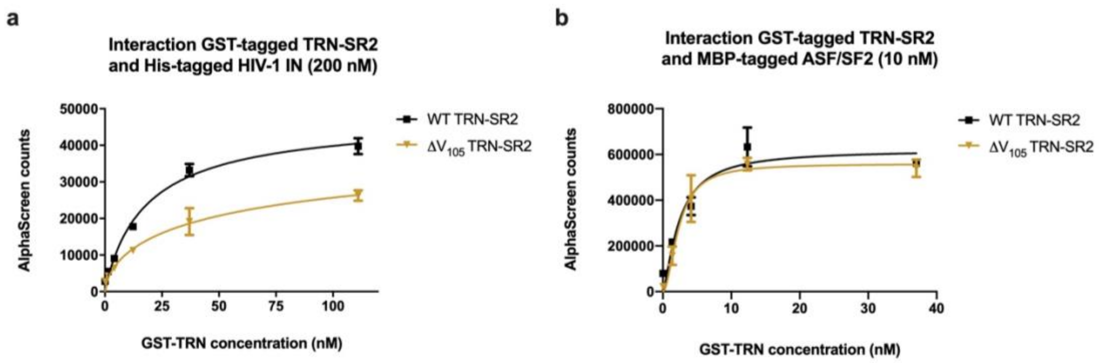

AlphaScreen-based analysis of the interaction between **(a)** GST-tagged WT TRN-SR2 or GST-tagged ΔV_{105} TRN-SR2 (corresponding to TRN-SR2 expressed in HeLaP4#15.15) with HIS_6-tagged integrase (IN). TRN-SR2 protein was titrated over a fixed concentration of HIV-1 IN (200 nM). **(b)** GST-tagged WT TRN-SR2 or GST-tagged ΔV_{105} TRN-SR2 with MBP-tagged ASF/SF2. TRN-SR2 protein was titrated over a fixed concentration of ASF/SF2 (12 nM). Mean and standard deviation are shown from one representative experiment, performed in duplicate, of two experiments.
